# Mechanical properties of tubulin intra- and inter-dimer interfaces and their implications for microtubule dynamic instability

**DOI:** 10.1371/journal.pcbi.1007327

**Published:** 2019-08-30

**Authors:** Vladimir A. Fedorov, Philipp S. Orekhov, Ekaterina G. Kholina, Artem A. Zhmurov, Fazoil I. Ataullakhanov, Ilya B. Kovalenko, Nikita B. Gudimchuk

**Affiliations:** 1 Department of Biology, Lomonosov Moscow State University, Moscow, Russia; 2 Moscow Institute of Physics and Technology, Dolgoprudny, Russia; 3 Sechenov University, Moscow, Russia; 4 Department of Physics, Lomonosov Moscow State University, Moscow, Russia; 5 Center for Theoretical Problems of Physicochemical Pharmacology, Russian Academy of Sciences, Moscow, Russia; 6 Federal Research and Clinical Center of Specialized Medical Care and Medical Technologies, Federal Medical and Biological Agency of Russia, Moscow, Russia; 7 Astrakhan State University, Astrakhan, Russia; 8 Peoples’ Friendship University of Russia (RUDN University), Moscow, Russia; King’s College London, UNITED KINGDOM

## Abstract

Thirteen tubulin protofilaments, made of αβ-tubulin heterodimers, interact laterally to produce cytoskeletal microtubules. Microtubules exhibit the striking property of dynamic instability, manifested in their intermittent growth and shrinkage at both ends. This behavior is key to many cellular processes, such as cell division, migration, maintenance of cell shape, etc. Although assembly and disassembly of microtubules is known to be linked to hydrolysis of a guanosine triphosphate molecule in the pocket of β-tubulin, detailed mechanistic understanding of corresponding conformational changes is still lacking. Here we take advantage of the recent generation of in-microtubule structures of tubulin to examine the properties of protofilaments, which serve as important microtubule assembly and disassembly intermediates. We find that initially straight tubulin protofilaments, relax to similar non-radially curved and slightly twisted conformations. Our analysis further suggests that guanosine triphosphate hydrolysis primarily affects the flexibility and conformation of the inter-dimer interface, without a strong impact on the shape or flexibility of αβ-heterodimer. Inter-dimer interfaces are significantly more flexible compared to intra-dimer interfaces. We argue that such a difference in flexibility could be key for distinct stability of the plus and minus microtubule ends. The higher flexibility of the inter-dimer interface may have implications for development of pulling force by curving tubulin protofilaments during microtubule disassembly, a process of major importance for chromosome motions in mitosis.

## Author summary

The ability to self-assemble from tubulin dimers in presence of guanosine triphosphate (GTP) and spontaneously disassemble, when GTP molecules in tubulin pockets are hydrolyzed, is a dramatic and essential feature of microtubules. This behavior has many important roles, including chromosome segregation in mitosis, rapid remodeling of the microtubule networks, establishing cell polarity and other. Nevertheless, the mechanism, linking the associated nucleotide with the conformational changes in tubulins, remains elusive. Most studies suggested that the nucleotide should affect either the equilibrium shape of tubulin dimers or the strengths of the lateral bonds between them. But existing experimental methods have lacked spatio-temporal resolution to test that. Theoretical studies, until recently, have suffered from the absence of high-resolution microtubule structures with different nucleotides to build on and the lack of computational efficiency to examine large tubulin assemblies. Here we use recent cryo electron microscopy structures of GDP and GTP-like microtubules, and employ all-atom molecular dynamics simulations to examine tubulin protofilaments. We find that the nucleotide primarily affects the interface between two tubulin dimers, making it more flexible in the GTP state. This makes the GTP-bound tubulin protofilament easier to incorporate into microtubule lattice, providing a simple mechanism for microtubule dynamic instability.

## Introduction

αβ-tubulin heterodimers polymerize into microtubules, hollow cylindrical structures, usually composed of 13 laterally attached protofilaments [1]. Microtubules are about 25 nm wide and range in lengths from tens up to millions of nanometers. They form cilia and flagella and serve as tracks for long-distance transport of intracellular cargos, such as vesicles and organelles. In contrast to other polymers, microtubules are highly non-equilibrium systems [2], which can remain in growth and shrinkage phases with relatively rare spontaneous transitions between them [3]. Because of this behavior, known as dynamic instability, individual microtubules display significant length changes, even at steady state. They elongate at their tips by addition of guanosine triphosphate (GTP)-bound tubulins. Soon after incorporation into microtubule lattice, GTP molecules are hydrolyzed to guanosine diphosphate (GDP). This leads to a conformational change in tubulins, so the lattice made of GDP-tubulins becomes less stable and more prone to depolymerization. However, because of a lag between the association of GTP-tubulins with microtubules and GTP hydrolysis, there is a certain number of GTP-tubulins at the growing microtubule tip, known as GTP cap, which prevents disassembly until the stabilizing cap is lost [4]. Both ends of microtubules are dynamically unstable. The end, exposing β-tubulin subunits, is called the plus-end. It grows faster than the other end, known as the minus-end. The origin of the difference of behavior between the plus and minus-ends of microtubules is currently poorly understood. In cells, the minus-ends are usually capped, so they remain stable. The plus ends are usually dynamic and serve multiple roles. During mitosis they generate forces responsible for chromosome motions, leading to segregation of duplicated DNA between daughter cells [5,6]. This fact has been extensively exploited for therapeutics, as the inhibition of microtubules dynamics by small molecule drugs leads to arrest of cell division followed by apoptosis, leading to a powerful method to fight proliferation of actively dividing tumor cells [7].

Despite extensive studies of dynamic instability for over three decades, the molecular features of GTP- and GDP-tubulins, determining their distinct propensity to polymerize, remain unclear. Early cryo electron microscopy (EM) studies reported very distinct shapes at the ends of growing and shortening microtubules [8]. That observation informed a so called ‘allosteric’ model of the GTP cap, postulating that GTP hydrolysis induced an allosteric conformational change in straight GTP-tubulin dimers, so GDP-tubulin became curved. Further cryo EM work modified the allosteric model, proposing that GTP-tubulin was also slightly curved, but still straighter than GDP-tubulin. The latter modification was based on observations of gently curving extensions on growing microtubule tips [8,9], and the shapes of tubulin structures formed in presence of slowly hydrolysable GTP analogue, GMPCPP [10,11].

Subsequent studies have accumulated substantial evidence indirectly supporting an alternative, ‘lattice’ model of the GTP-cap, postulating that the phosphorylation state of tubulin-bound nucleotide affects the strengths of inter-tubulin bonds, while the shapes of free GTP- and GDP-bound tubulins remain similar. The following lines of evidence against a significant difference in curvature between GTP- and GDP tubulins have been reported: (1) free tubulin dimers and tetramers are similarly curved in all available crystal structures of tubulin (reviewed in [12]); (2) there is no significant difference in the shape of GTP- and GDP-tubulins according to small-angle X-ray scattering measurements [13]; (3) affinity to allocolchicine, which is thought to be able to bind only a curved intra-dimer interface, is the same for GTP- and GDP-tubulins [13]; (4) recent cryo electron tomography reveals essentially no difference in the curvatures of protofilaments at the tips of growing and shortening microtubules [14]. Finally, computational studies so far have consistently found that the relaxed tubulin conformation is curved, irrespective of the nucleotide bound, while the strengths of the inter-tubulin bonds are likely to be nucleotide-dependent [15–21]. We note, however, that at the time when many of those important pioneering simulations were carried out, no high resolution structures of both GTP- and GDP-tubulins were yet available. The first relatively high resolution cryo-EM-based structures of tubulins in microtubule walls in presence of GDP or GTP analog, GMPCPP, were presented in the seminal paper of Alushin et al., 2014 [22]. But even those structural data and their subsequent improvements have not yet produced a fully consistent picture that would clearly support only the allosteric or the lattice model. For example, reports from the Nogales group did not detect any considerable changes at the lateral tubulin-tubulin interfaces and emphasized the “compaction” and skew of GDP- tubulins, in contrast to “extended” GTP-tubulin state in the lattice [22–24]. The authors proposed that compaction could induce internal mechanical strain in GDP-tubulin. A study from Moores’ group, however, suggested that lateral bonds were not unchanged, but weakened after GTP hydrolysis [25].

These controversies, together with the inability of modern structural methods to directly visualize conformational changes of tubulins following GTP hydrolysis and breakage of lateral bonds have encouraged us to undertake a new computational study, in which we re-investigate the effects of nucleotides on the shape and mechanics of tubulins, taking advantage of the wealth of newly available structural data and new computational resources. A recent cryo electron tomography work indicated importance of tubulin protofilaments, rather than just free dimers, as structural intermediates of microtubule assembly process [14]. Therefore, both intra-dimer and inter-dimer interfaces could have an important role in dynamic instability. To take this into account, we carried out molecular dynamics simulations of tubulin protofilaments, which contained both types of inter-tubulin interfaces. The tubulin protofilaments were extracted from the microtubule wall in compacted, GDP-bound, or extended, GTP-bound, states. In our simulations, the tubulin protofilaments in each of these nucleotide states relaxed to similar non-radially curved and twisted conformations, in contrast to the expectations of the allosteric model of microtubule instability. Our further analysis suggested that GTP hydrolysis primarily affected the flexibility and conformation of the inter-dimer interface, without a strong impact on the shape or flexibility of αβ-tubulin heterodimer. The inter-dimer interfaces of GTP-tubulins were significantly more flexible than those of intra-dimer interfaces. We argue that such a difference in flexibility could be key for distinct dynamic behavior of plus and minus microtubule ends.

## Results

### Straight GTP- and GDP- tubulins relax to similar non-radially bent conformations

**Fig 1:**
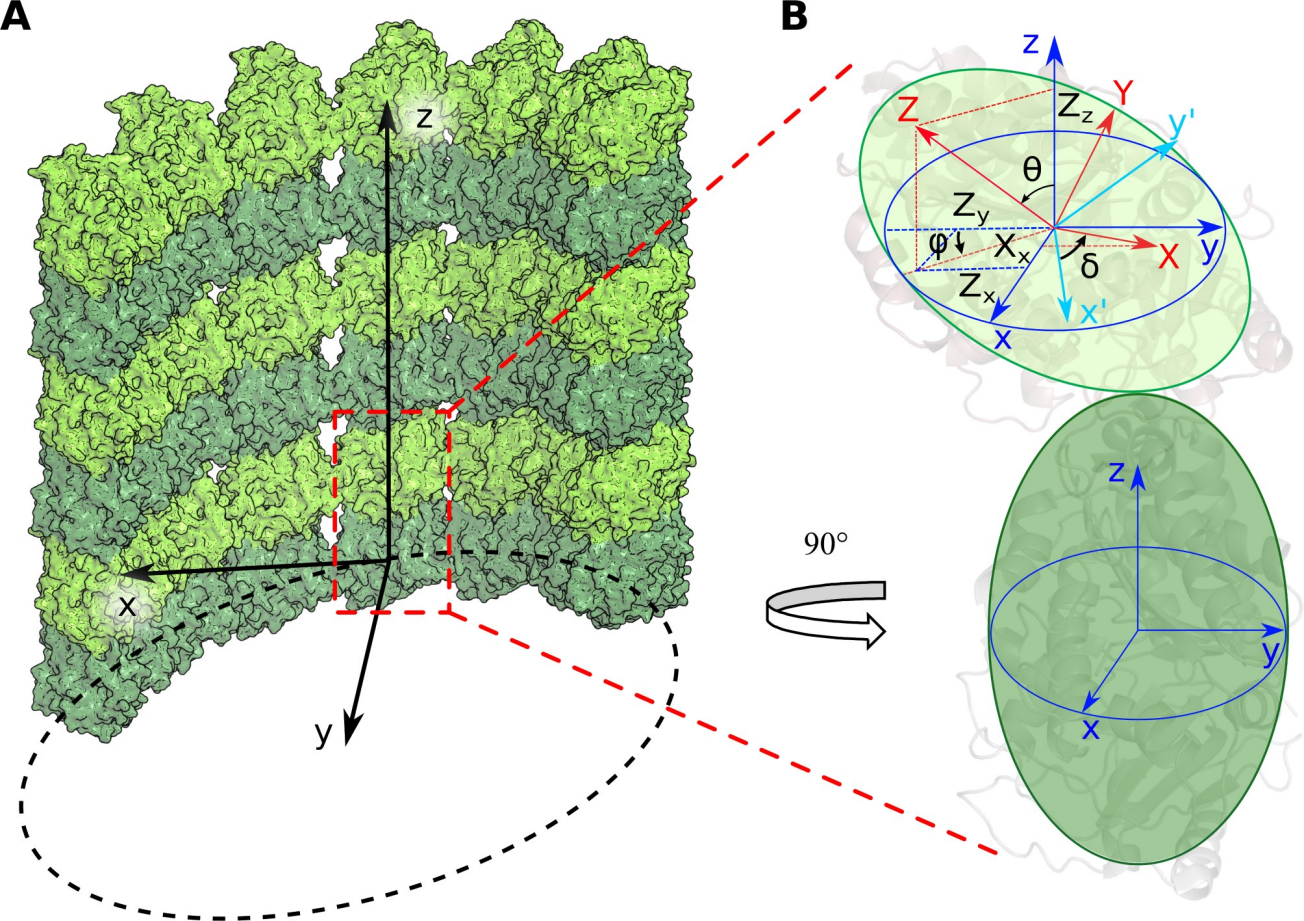
Angles describing the rotation of monomers relative to each other. A: Microtubule-bound coordinate system *xyz*. α-tubulins are shown in dark green, β-tubulins in light green. *x*-axis is radial to the microtubule axis, *y*-axis is tangential to microtubule, *z*-axis is parallel to microtubule axis. B: Schematic representation of a curved tubulin dimer and the angles that characterize the magnitude of upper subunit tilt, *θ*, the direction of this tilt relative to the radius of the microtubule, *φ*, and the twist angle *δ* of tubulin monomers with respect to one another. The coordinate system *xyz* associated with the bottom monomer is shown in blue, the system *XYZ*, associated with upper monomer, is red. Cyan is an auxiliary coordinate system *x'y'z'*, which is produced by rotation of the coordinate system *xyz*, so the vector *oz* becomes aligned with OZ.

To characterize the nucleotide dependence of tubulin’s shape in relaxed tubulin dimers and short tubulin protofilaments, we prepared all-atom molecular dynamics models of tubulins, extracted from cryo-EM-based structures of GDP- and GTP-like (GMPCPP) microtubule lattice [22]. Flexible tubulin tails were included in the simulation to make sure that their potential effects on tubulin conformations would be taken into account [26]. For each nucleotide, we carried out two one-microsecond-long simulations of tubulin dimers, and three one-microsecond-long simulations of short protofilaments, representing two longitudinally bonded dimers (S1 and S2 Movies). In order to put the resulting conformational changes of tubulins into the context of a microtubule, we aligned the α-tubulin subunits of each simulated structure with a microtubule wall fragment, so all types of rotations could be assessed relative to the microtubule-bound coordinate system xyz (Fig 1).

**Fig 2:**
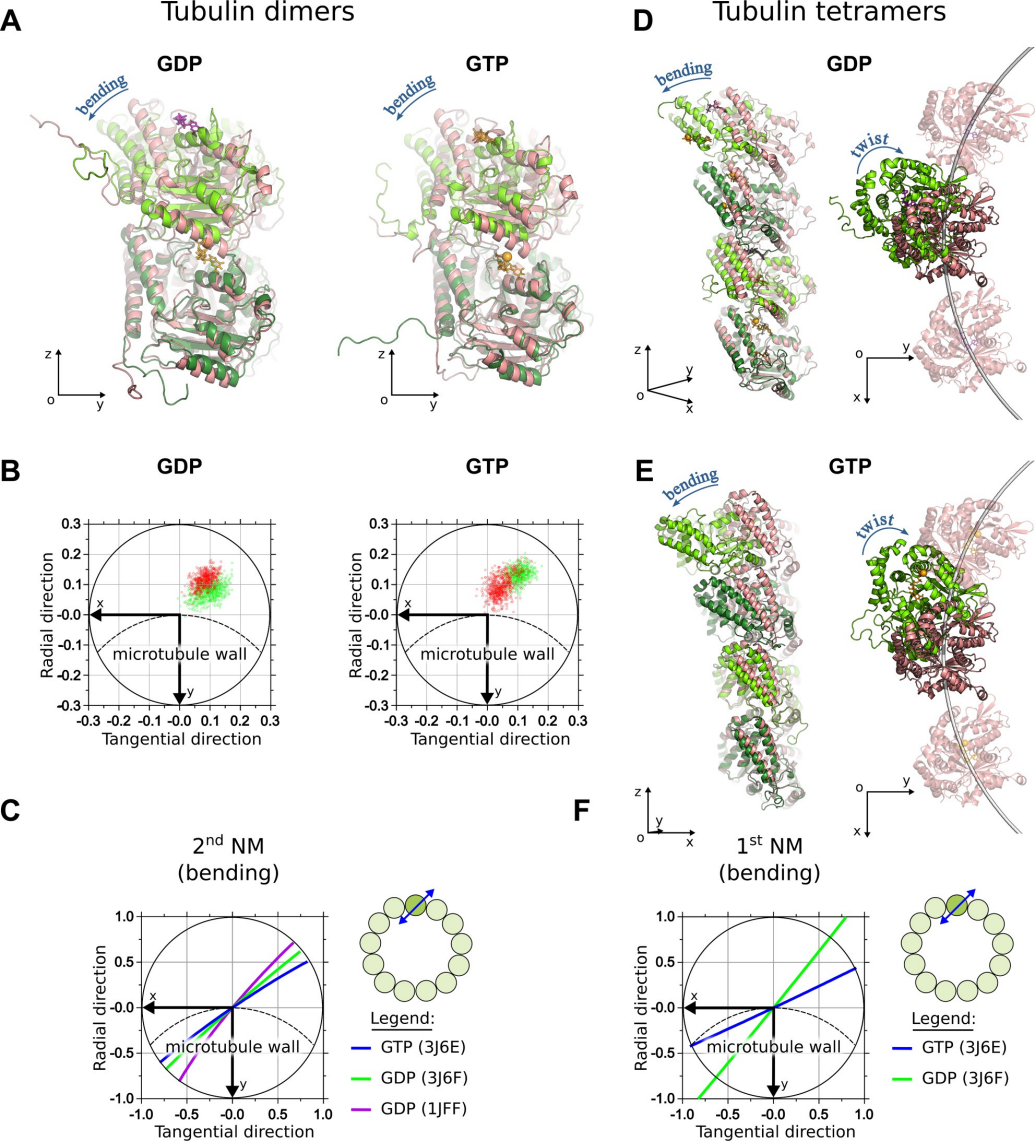
Simulations of GDP- and GTP-tubulin dimers. A: GDP- and GTP-tubulin dimers at the end of 1 μs simulation (green) aligned onto initial straight structures (pink). GTP is shown in orange, GDP is purple. B: Projections of the unit OZ-vector of the β-subunit of GDP-tubulin dimer onto *xy*-plane of the α-tubulin at every nanosecond of the simulation after the first 500 ns. Dashed line schematically shows the circumference of the microtubule. Horizontal axis is tangential to the microtubule, vertical axis is directed radially toward microtubule axis. Green and red data points correspond to two different independent simulation runs. C: Projections of the center of mass of β-tubulin onto xy-plane of the α-tubulin during the motions, corresponding to major bending mode of free tubulin dimer. Lines of different colors mark different tubulin structures. Schematic in the upper right corner of this panel illustrate approximate direction of bending motions relative to the microtubule. D: GDP-tubulin tetramer in the end of 1 μs simulation (green) aligned onto initial straight structure (pink), shown in two orthogonal views: in the plain of bending (left) and along the microtubule axis (right). In the latter view, the subunits of the two adjacent protofilaments are also shown. E: Analogous views of GTP-tubulin tetramer. F: Projections of the center of mass of the top β-tubulin onto *xy*-plane of the bottom α-tubulin during the motions, corresponding to major bending mode of a free tubulin tetramer (also see S3 Movie).

Consistent with previous reports [15,21,27], in our simulations both GDP- and GTP-dimers relaxed over time to similar bent shapes (Fig 2A). Although the bending occurred predominantly in the ‘outward’ direction, it did not happen in the plane that contained the microtubule axis, as is clearly seen in Fig 2B, which shows projections of the unit orientation vector of the β-tubulin subunit relative to α-tubulin subunit. Additional molecular dynamics simulations, based on a GDP-tubulin dimer structure extracted from zinc-induced sheets (PDB code: 1JFF), indicated that such non-radial bending and a slight twist were common conformational changes shared by other structures of tubulin (S1A and S1B Fig).

To independently validate the tendency of dimers to curve in non-radial plane, we predicted major low-frequency modes of motion for three tubulin dimer structures, using normal modes analysis (NMA). The first NM in all types of tubulin dimer structures examined corresponded to predominantly twisting motions of β-tubulin relative to the α-tubulin subunit, while the second and the third NMs represented predominantly non-radial bending, similar to a previous report [28].

Major low-frequency modes of GTP- and GDP-tubulin dimers were very similar. Principal component analysis (PCA) of molecular dynamics simulation trajectories identified significant correlation of the major NMs with principal components (PCs) of motions in molecular dynamics. The first three PCs explained, on average, 65% of observed tubulin dimer motions (S1 Table). Overall, the major bending motions were represented by the second NM (Fig 2C). They occurred in a direction very similar to that of the major conformational changes observed in our molecular dynamics simulations (Fig 2B).

Conformational analysis of tubulin tetramers revealed that additional inter-dimer interface did not qualitatively change the overall fashion of bending of the whole tetramer, compared to that of a tubulin dimer. Specifically, both GDP- and GTP-tubulin tetramers also assumed outwardly curved shapes in the end of one-microsecond-long simulations (Fig 2D and Fig 2E). The overall bending was also non-radial. Likewise, the dominant NM of the whole tetramer corresponded to bending in a similar non-radial direction (Fig 2F, S3 Movie) and it overlapped significantly with PCs of motions in molecular dynamics (S2 Table). The first three PCs explained, on average, 79% of the total variance present in the molecular dynamics trajectories of tetramers (S2 Fig).

### Tubulin conformation is more nucleotide-sensitive at the inter-dimer interfaces than at the intra-dimer interfaces

To gain more detailed insight into the conformational changes at intra- and inter-dimer tubulin interfaces during their relaxation from straight to curved shapes and their dependence on the associated nucleotide, we described the relative motions of adjacent tubulin monomers at each interface with three rotation angles, using metrics similar to those introduced previously [16]. Specifically, bending at the tubulin-tubulin interface was described with two angles: *θ-*angle characterized the magnitude of the conformational change, while auxiliary angle, *φ*, showed the direction in which the bending occurred; *δ*-angle characterized twist of one tubulin monomer relative to the other (see Methods for more details). To determine the direction of rotations relative to the microtubule structure, we aligned the minus-end-proximal tubulin monomer onto a corresponding subunit in a straight microtubule fragment, which was oriented relative the coordinate system as depicted in Fig 1. In this arrangement, outward strictly radial bending is described by positive *θ*-angles and *φ* = 0 degrees. Despite the advantage of being physically clear and easy to relate with microtubule geometry and with the degrees of freedom, which are usually present in higher-scale models of microtubule dynamics, the rotation angles may not optimally represent the multi-dimensional molecular dynamics data. For this reason, we also carried out an additional analysis, projecting molecular dynamics trajectories on two main PCA modes and comparing the movements at tubulin interfaces, expressed in those observables (S3 Fig, S4 Fig, S4 Movie). This yielded essentially similar conclusions about the relative properties of the inter- and intra-dimer interfaces, so we decided to stick to rotation angles throughout this report for the sake of intuitiveness and physical clarity of description.

**Fig 3:**
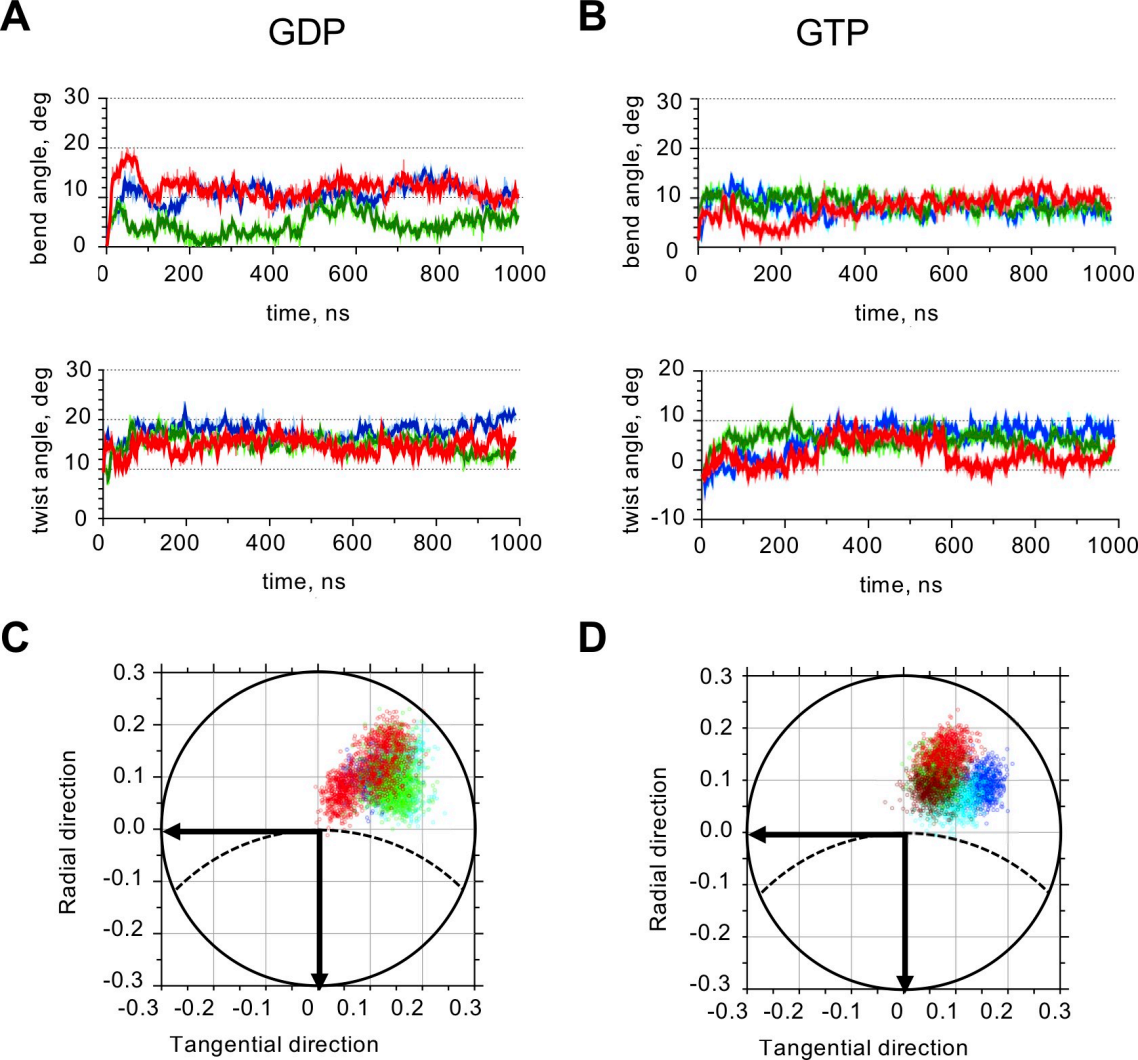
Quantitative analysis of intra-dimer interface of GTP- and GDP-tubulin tetramers. A: Time-dependence of GDP-tubulin intra-dimer bend and twist angles. Colors mark independent simulation runs. Only one of the two intra-dimer interfaces for each tetramer simulation run is shown. B: Time-dependence of GTP-tubulin intra-dimer bend and twist angles. Colors mark independent simulation runs. C: Projections of the unit OZ-vector of the β-subunit of GDP-tubulin dimer onto the xy-plane of the α-tubulin at every ns after the first 500 ns of the simulation. Data and color-coding correspond to panel A. Dashed line schematically shows the circumference of the microtubule. Horizontal axis is tangential to the microtubule, vertical axis is directed radially toward microtubule axis. D: Projections of the unit OZ-vector of the β-subunit of GTP-tubulin dimer onto xy-plane of the α-tubulin at every ns after the first 500 ns of the simulation. Data and color-coding correspond to panel B.

First, we calculated rotation angles in tubulin tetramers at intra-dimer tubulin interfaces. After 500 ns of simulation, intra-dimer interfaces of both GDP- and GTP-tubulin tetramers relaxed to a conformation, in which their β-subunits were tilted relative to α-tubulins with almost identical magnitudes of intra-dimer bending, 9.4 ± 0.9 and 8.2 ± 0.7 degrees, respectively (Fig 3, Table 1). Simultaneously, β-tubulins twisted relative to α-tubulins by about 5.2 ± 0.9 and 7.0 ± 1.5 degrees, in GDP- and GTP- states. Conformational changes of free dimers at the intra-dimer interface were quantitatively similar (Table 1, S5 Fig).

**Table 1:** Quantification of conformational changes at intra- and inter-dimer interfaces in molecular dynamics simulations.

PDB id	Nucleotide	Interface	Direction of bending (φ), deg	Bend angle (θ), deg	Twist angle (δ), deg	Number of runs	Total simulation time, μs
**Tubulin dimers**							
3J6F	GDP	intra	-44 ± 8	7.8 ± 0.5	9.4 ± 2.4	2	2
3J6E	GTP	intra	-33 ± 6	7.8 ± 2.0	5.1 ± 0.8	2	2
**Tubulin tetramers**							
3J6F	GDP	intra	-48 ± 4	9.4 ± 0.9	5.2 ± 0.9	6	6
3J6E	GTP	intra	-40 ± 6	8.2 ± 0.7	7.0 ± 1.5	6	6
3J6F	GDP	**inter**	-141 ± 35	5.2 ± 1.7	4.4 ± 3.0	3	3
3J6E	GTP	**inter**	-73 ± 22	9.1 ± 0.4	7.7 ±5.6	3	3

All values represent mean ± standard deviation based on data from the second half of the simulations (after 500 ns). Values for intra-dimer interfaces in tetramers combine data from two intra-dimer interfaces, hence their doubled effective number of runs and simulation time.

**Fig 4:**
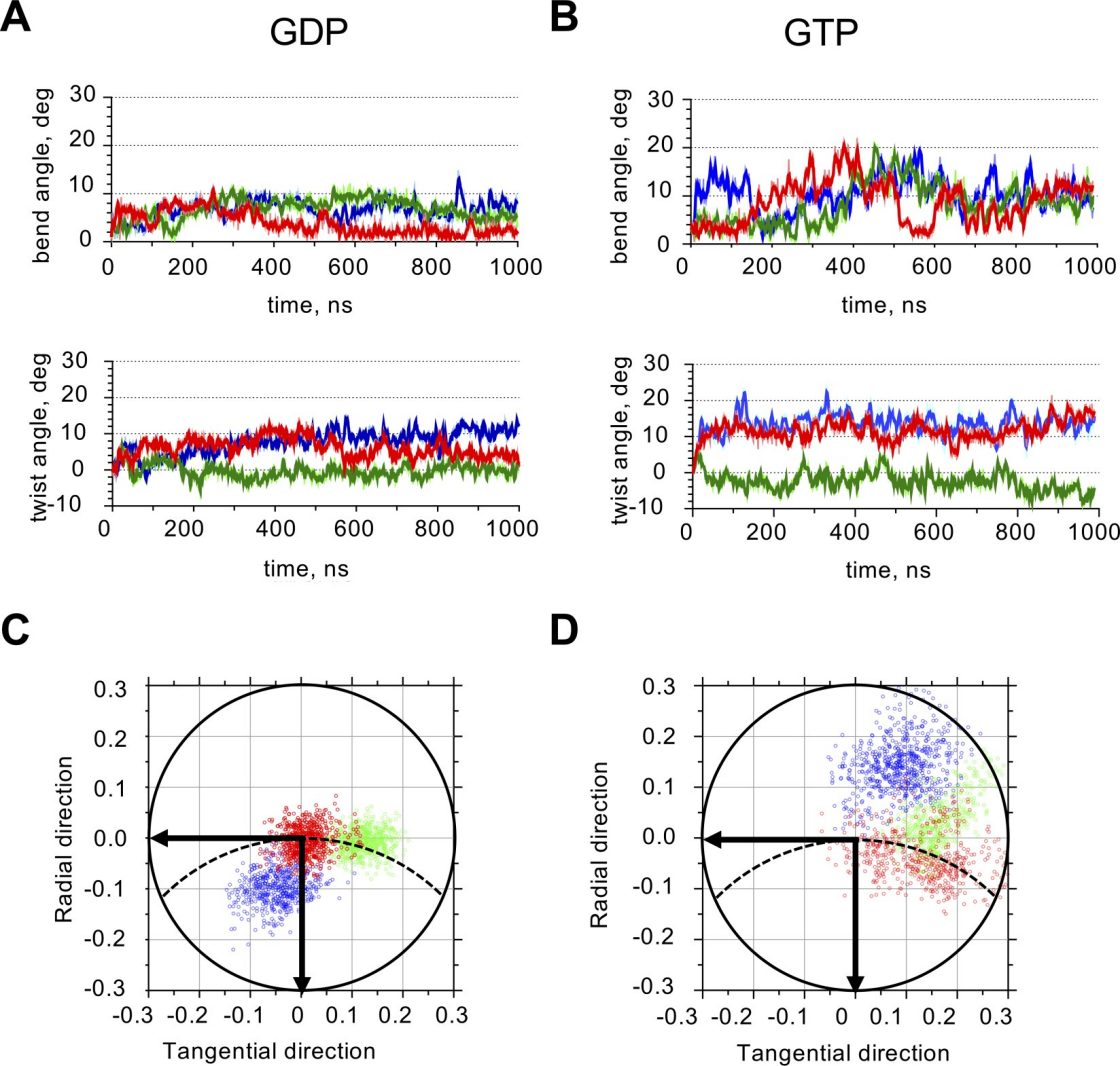
Quantitative analysis of inter-dimer interface of GTP- and GDP-tubulins. A: Time-dependence of GDP-tubulin inter-dimer bend and twist angles. Colors mark three independent simulation runs. B: Time-dependence of GTP-tubulin inter-dimer bend and twist angles. Colors mark three independent simulation runs. C: Projections of the unit OZ-vector of the α-subunit of the GDP-tubulin inter-dimer interface onto the xy-plane of the β-tubulin at every ns after the first 500 ns of the simulation. Data and color-coding correspond to panel A. Dashed line schematically shows the circumference of the microtubule. Horizontal axis is tangential to the microtubule, vertical axis is directed radially toward microtubule axis. D: Projections of the unit OZ-vector of the α-subunit of GTP-tubulin inter-dimer interface onto xy-plane of the β-tubulin at every ns after the first 500 ns of the simulation. Data and color-coding correspond to panel B.

In contrast to intra-dimer interfaces, the inter-dimer interfaces were less reproducible in their bending directions from run to run, suggesting the presence of multiple local minima in the energy landscape (Table 1, Fig 4). Within each run, the bending angle had a satisfactory convergence to a stable mean value, as assessed by splitting the last 500 ns of the simulations in four 125-ns-long segments and analyzing them separately (S7 Fig).

We compared equilibrium conformational angles of tubulins at the end of individual simulation runs with the respective intra- and inter-dimer angles calculated for published crystal structures of tubulins bound to microtubule associated proteins (MAPs): stathmin, darpin, TOG-domain or MCAK proteins [29–34]. Intra-dimer curvature, its direction and magnitude of twist in simulated structures were similar to characteristics of the experimental structures, confirming the ability of molecular dynamics simulations to predict equilibrium shapes of tubulin dimers (Table 2, S1C Fig). Inter-dimer angles, though, were much more variable in simulations, without clear correlation with the corresponding angles in crystallized tubulin-MAP complexes. We speculate that in complex with a MAP, the tubulin tetramer is likely to be fixed by its interaction partner, resulting in a different direction of inter-dimer bending and in lower flexibility of the inter-dimer interface. Hence, the conformational variability is reduced.

**Table 2:** Quantification of conformational angles at intra- and inter-dimer tubulin interfaces in published structures.

PDB id	Nucleotide	Interface	Direction of bending (φ), deg	Bend angle (θ), deg	Twist angle (δ), deg	MAPs and ligands in complex	reference
3RYF	GTP	intra	-28.0	9.0	5.9	stathmin	[29]
		**inter**	-32.7	9.9	-8.5		
3RYI	GDP	intra	-29.9	9.6	6.1	stathmin	[29]
		**inter**	-35.3	9.2	-8.9		
1SA0	GDP	intra	-35.9	10.1	7.0	Stathmin, colchicine	[30]
		**inter**	-35.1	11.0	0.7		
4DRX	GTP	intra	-27.2	10.1	5.2	darpin	[31]
4FFB	GTP	intra	-38.6	11.7	7.1	Stu2 TOG1	[32]
4U3J	GTP	intra	-41.4	11.5	4.3	Stu2 TOG2	[33]
5MIO	GDP	intra	-40.1	13.1	6.1	darpin, MCAK, colchicine	[34]

### Attachment to the microtubule tip may affect tubulin oligomer conformation due to constraints imposed by the microtubule lattice

**Fig 5:**
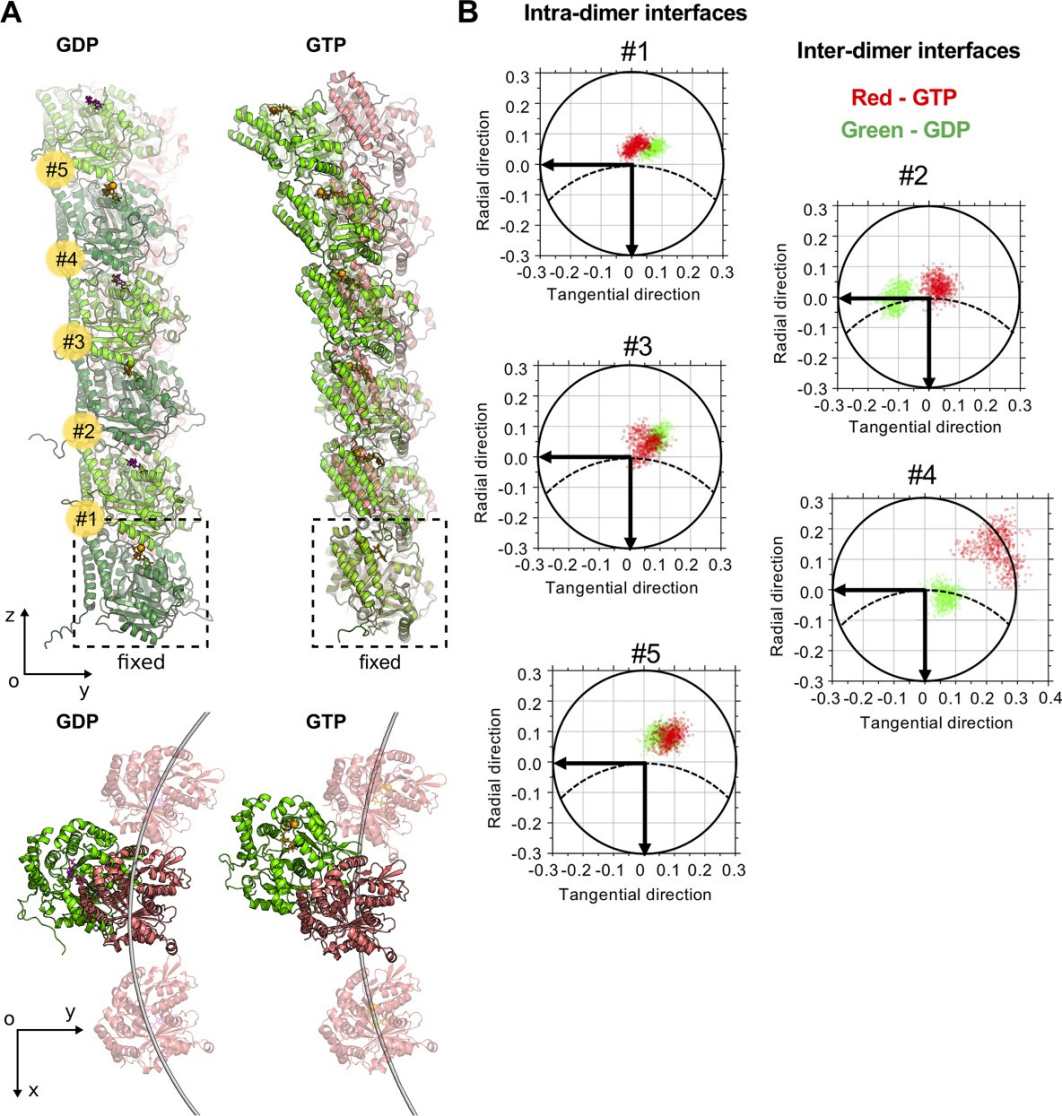
Effects of attachment to mechanical support on tubulin oligomer conformation. A: GDP- and GTP-tubulin hexamers at the end of 1 μs simulation (green) aligned onto initial straight structures (pink), viewed from the side and from top. GTP molecule and Mg^2+^ ion are shown in orange, GDP molecule is purple. Five inter-tubulin interfaces are numbered from bottom to top (in yellow circles). B: Projections of the unit OZ-vector of the upper tubulin subunit at each tubulin interface onto xy-plane of the lower tubulin at every nanosecond of the simulation after the first 500 ns. Dashed line schematically shows the circumference of the microtubule. Horizontal axis is tangential to the microtubule, vertical axis is directed radially toward microtubule axis. Green and red data points correspond to GDP- and GTP-tubulin data, respectively. Interfaces are numbered as in panel A.

Despite the fact that in our simulations and in experimental structural data tubulin oligomers both display non-radially curved and twisted shapes, recent cryo electron tomography studies reported nearly planar protofilaments at the tips of growing and shortening microtubules [14,35]. Puzzled by this discrepancy, we performed additional molecular dynamics simulations of GTP- and GDP-tubulin hexamers, applying position restrain on Cα atoms of the minus-end proximal tubulin subunit (Fig 5A, S4 Movie). Fixation of the terminal α-tubulin was mimicking oligomer attachment to the microtubule end. Analysis of the relaxed shapes of GTP- and GDP-tubulins after 500 ns simulation revealed that the interfaces proximal to the fixed subunit (#1–3) tended to be overall straighter, less flexible, and bent in a more radial direction (Fig 5B). On the other hand, distal interfaces (#4 and #5) behaved essentially like those of free tubulin oligomers (compare with Fig 4C, Fig 4D, Fig 3C and Fig 3D). Given this ‘straightening’ effect due to the longitudinal attachment to the plus tip, overall bending direction of whole hexamers, characterized by projections of the center of mass of β-tubulin subunit onto XY-plane, was somewhat more radial on the scale of hexamers, compared with free tetramers (Fig 5A, top view). Although, we note that at the scale of longer protofilaments, the tangential component may still be significant.

**Fig 6:**
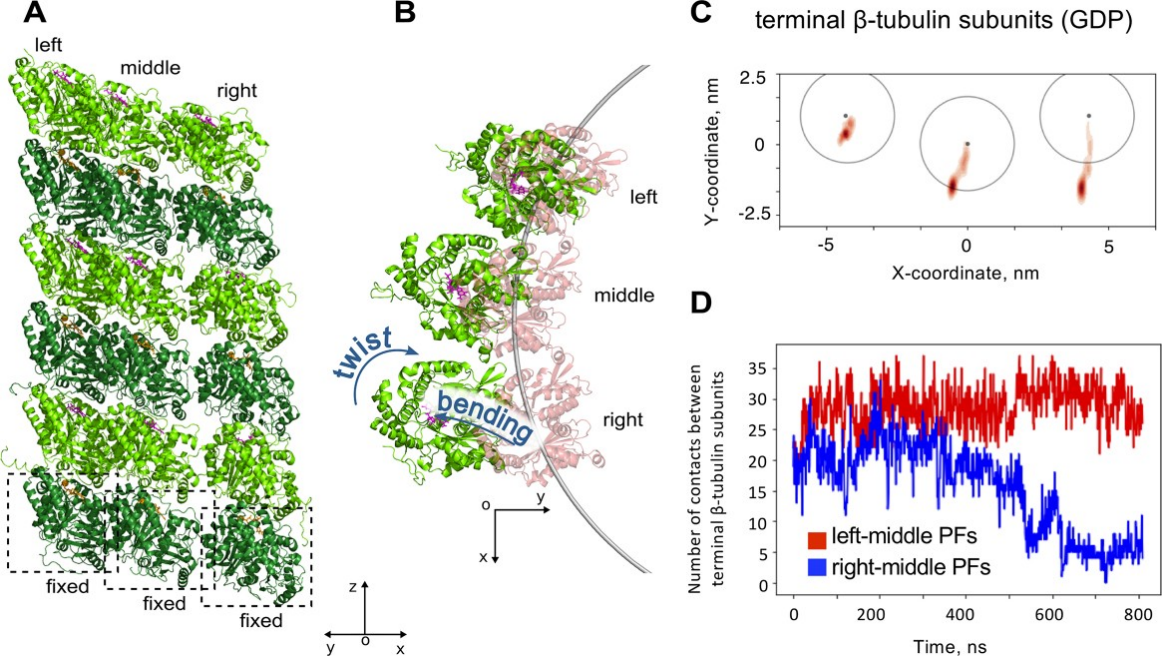
Effects of lateral protofilament neighbors on tubulin oligomer relaxation from straight conformation. A: three GDP-tubulin hexamers after 0.8 μs simulation, viewed in diagonal direction. B: the same structure as in panel A, viewed from top. C: Smoothed projections of the centers of mass of terminal β-tubulin subunits onto XY plane of microtubule-bound coordinate system. D: Number of contacts between Cα-atoms of the terminal β-tubulin subunits during one the simulation run. Red curve corresponds to contacts between the left and the middle protofilaments, blue curve is for contacts between the middle and the right protofilaments.

We also hypothesized that the presence of adjacent protofilament neighbors could affect the bending direction of a given protofilament, attached to the microtubule tip. To test that, we constructed a molecular model of three laterally bound GDP-tubulin hexamers, whose minus-end proximal α-tubulins were fixed (Fig 6A, S5 Movie). In two independent simulations of this system, the whole assembly of three protofilaments consistently bent asymmetrically: the splaying amplitude of the right protofilament was the highest, while the left protofilament remained almost straight (‘left’ and ‘right’ are defined as in Fig 6A and Fig 6B). This result can be explained by the tendency of individual protofilaments to bend and twist in the directions depicted in Fig 6B, Fig 2D and Fig 2E. Such motions tend to stretch the left lateral bond significantly less, compared to the right bond. Therefore, it is the right lateral bond, which restricts the motion more significantly. Hence, in the absence of the right lateral bond the right protofilament undergoes relaxation to the bent and twisted state relatively easily, while the left protofilament is almost fully restricted by its right lateral bond (Fig 6B and Fig 6C). As a result, the splaying amplitude of the right protofilament is a lot more dramatic. This splaying leads to breakage of the lateral bond between the terminal β-tubulin subunits of the right and the middle protofilaments during the simulation, while the bond between the terminal β-tubulin subunits of left and the middle protofilaments remained intact, as we verified by counting the number of contacts between amino acids of those subunits (Fig 6D). Comparison of protofilament bending in the simulations in the presence or absence of adjacent protofilaments did not reveal a marked effect of lateral neighbors on the bending direction of the middle protofilament (S8 Fig).

### Inter-dimer interface of GTP-tubulin is more flexible than its intra-dimer interface, and it stiffens upon GTP hydrolysis

We noticed that the variance of rotation angles at the inter-dimer GTP-interface was higher than at all other types of interfaces, which can be appreciated by the highest scatter of projections of the unit OZ-vector, characterizing direction and amplitude of tubulin bending (Fig 4D vs. Fig 4C, Fig 3C, Fig 3D). Similar increased variance is seen at the inter-dimer GTP-interface, when the data are examined by projecting on the two main PCA modes (S3 Fig, S4 Fig, S4 Table, S5 Table). Guided by this observation, we hypothesized that the nucleotide could have a distinct effect on the mechanical properties of the inter-dimer interface. We therefore used two methods to quantify the flexural stiffness of tubulin interfaces. The first method was based on the equipartition theorem. Assuming that tubulin structures were already equilibrated by 500 ns of simulation, we pooled tubulin angles after that time and calculated their variance. According to equipartition theorem, at thermodynamic equilibrium variance, *σ*^2^, of a given conformational angle (*θ* or *δ*) should be inversely proportional to the respective harmonic flexural stiffness *κ*:

$$\kappa =\frac{k_{B}T}{\sigma^{2}}$$ (1)

where *k*_*B*_ is the Boltzmann constant, *T* is the temperature. Resulting harmonic stiffness values are summarized in Table 3.

**Table 3:** Harmonic stiffness of tubulin conformational angles, k_B_T/rad^2^.

PDB id	Nucleotide	Interface	Bending stiffness	Twisting (torsional) stiffness
3J6F	GDP	intra	930 ± 120	990 ± 150
3J6E	GTP	intra	1100 ± 120	1160 ± 100
3J6F	GDP	**inter**	1290 ± 510	760 ± 60
3J6E	GTP	**inter**	350 ± 110	410 ± 70

These results suggest that the inter-dimer interface of GTP-tubulin is considerably more flexible than its intra-dimer interface in all kinds of rotation. Intra-dimer stiffnesses are not sensitive to nucleotide, speaking against the presence of significant allosteric effects of GTP hydrolysis on mechanical properties of the intra-dimer interfaces at equilibrium. Inter-dimer stiffnesses, however, are significantly lower in the GTP-tubulin model compared to the GDP-tubulin. The latter finding is suggestive of at least a partial contribution of flexural stiffness modulation from the nucleotide hydrolysis state into the mechanism of dynamic instability.

The equipartition theorem-based method has allowed comparing stiffnesses, corresponding to motions in a relatively narrow high-frequency range. To identify stiffnesses, characterizing conformational changes at longer timescales, we used NMA as a complementary approach. The squared mode frequency, related to each normal mode, can be reckoned into mechanical properties, such as bending stiffness/torsional rigidity, corresponding to the motion along the given mode [36–38]. As illustrated by Table 4, NMA confirmed that inter-dimer interface was much more flexible than the intra-dimer interfaces in the GTP-state, but that was not true for the inter-dimer interface of GDP-tubulin tetramer.

**Table 4:** Bending stiffness, *K*_*bend*_, and torsional rigidity, *K*_*twist*_, of intra- and inter-tubulin interfaces, inferred from NMA.

		Mode	1st (twist)	2nd (bend)
PDB id	Nucleotide	Interface	torsional rigidity, ×10^−28^ Nm^2^	bending stiffness, ×10^−28^ Nm^2^
3J6F	GDP	intra	2.99	4.57
3J6E	GTP	intra	1.78	3.97
3J6F	GDP	inter	3.82	6.56
3J6E	GTP	inter	0.99	1.62

It is tempting to hypothesize that significant stiffening of the inter-dimer interface may be caused by inter-dimer compaction of the GDP-tubulin. But such compaction could well be released upon relaxation of GDP-tubulin tetramer, extracted from microtubule lattice. So we questioned, whether or not free GTP- and GDP-tubulin tetramers converged in the simulations to conformations with similar extent of the inter-dimer compaction. For straight microtubule lattice, intra-dimer and inter-dimer distances were previously used to characterize the extent of tubulin compaction. They were defined as the lengths of the vectors, connecting ribose rings of the nucleotides in the corresponding pairs of longitudinally bonded tubulins [23]. Applying the same metric for our simulated tubulin tetramers and averaging over the second half of one-microsecond-long simulations, we found that both GTP- and GDP-tubulins displayed similar extended intra-dimer and similar shorter inter-dimers distances (S3 Table). Interestingly, these numbers closely matched the corresponding distances in crystal structures of curved tubulins, e.g. in the structure of tubulin tetramer in complex with stathmin and vinblastine (S3 Table, [30]).

However, we note that this metric should be used with caution for describing compaction of curved tubulin structures, because in this case, the inter-tubulin interfaces may not shrink or extend predominantly along the vector, connecting the nucleotides in adjacent tubulins. Moreover, other substantial conformational changes may be present. Therefore, we decided to additionally characterize the inter- and intra-dimer interfaces with the number of contacts between α- and β-tubulin amino acids at the interface. In fact, the number of contacts should correlate with compaction because amino acids at the more compact interface come closer together. We find that the inter-dimer interface of GDP-tubulin retains the largest number of contacts throughout the simulation (S6 Table). Thus, the high number of contacts at the GDP-tubulin inter-dimer interface might explain the enhanced flexural stiffness of this interface.

## Discussion

Taking advantage of the new generation of cryo-EM-based in-microtubule tubulin structures in GDP and GTP-like states, we carried out several one-microsecond-long molecular dynamics simulations of free tubulin dimers and tetramers. Our simulations reveal that initially “compacted” GDP-bound tubulins, and initially “extended” GTP-bound tubulins both adopt similar non-radially curved and slightly twisted shapes. The presence of substantial tangential bending and twist components in the resulting relaxed tubulin conformations, is fully consistent with published crystal structures of tubulins in complex with MAPs [29–34] (Tables 1 and 2). However, in contrast to those data, no pronounced out-of-plane bending was observed in a recent cryo electron tomography study, which reported essentially flat protofilaments, lying mainly in the radial planes, containing the microtubule axis [14,35]. The origin of this discrepancy is not completely clear. But it could be partially explained by the conformational effects, induced by attachment of tubulin oligomers to the plus-end of the microtubule, as suggested by our simulations of single and three tubulin hexamers, longitudinally fixed at the minus-end. It might also be possible that several longitudinally attached tubulins experience some kind of cooperative behavior, similar to described with FtsZ [39].

Strikingly, we find that the nucleotide type does not affect the curvature or mechanics of the intra-dimer interface, but it does considerably modify the stiffness of the inter-dimer interface. Prior to GTP hydrolysis the inter-dimer interface is significantly softer than the intra-dimer one. Based on our simulations, we propose that enlarged number of contacts at the inter-dimer interface in the GDP-state makes it even slightly stiffer than the intra-dimer interface.

In our opinion, these findings have at least three important implications for our understanding of the mechanisms of microtubule instability.

First, non-radial bending and twisting of tubulins during their relaxation from straight to curved configuration likely means that the lateral bonds on two sides of the splaying protofilaments at the microtubule tip experience unequal mechanical stress. This conclusion is qualitatively similar to the results of a previously study, which considered a hypothetical microtubule tip, constructed based on X-ray structures of curved tubulins in complex with stathmin [40]. We speculate that uneven distribution of mechanical stress on lateral bonds may lead to sequential, rather than simultaneous, rupture of the lateral bonds, which has not been previously considered by the majority of existing models of microtubule dynamics [41–46], with one notable exception [45]. This proposal is illustrated by our simulations of three laterally attached protofilaments, which splay apart by breaking lateral bonds between the right and the middle protofilaments sooner than the bonds between the middle and the left protofilaments (Fig 6). Given the fact that sequential breakage of the lateral bonds is energetically more feasible than their simultaneous breakage, we believe that taking this new feature of tubulin mechanics into account may lead to significant revision of current estimates for lateral bonds between tubulins. More work is needed to investigate full lateral bond rupture and its dependence on the nucleotide.

Second, stiffening of the compacted interface between GDP-tubulin dimers helps to resolve the apparent paradox of a lack of clearly visible effects of the nucleotide on lateral bonds and on the curvature of free tubulin protofilaments, despite a well-established link between microtubule stability and the type of nucleotides associated with the microtubule lattice. Indeed, most experimental work and theoretical thinking in the literature have been focused on an effort to explain dynamic instability by nucleotide-dependent curvature or by nucleotide-dependent strengths of lateral bonds. The possibility of modulating tubulin flexural stiffness by the nucleotide has been raised by only a small number of studies, e.g. [10,21,27], and underappreciated. Recently Igaev and Grubmüller suggested an allosteric mechanism for dynamic instability, based on the nucleotide-driven tubulin dimer stiffness change [21]. Our current study essentially points to a very similar idea: the softer interface between GTP-tubulin dimers requires less additional energy to straighten curved GTP-tubulin protofilaments in order to incorporate them into the microtubule wall, compared to a stiffer GDP-tubulin protofilament. However, importantly, in this study we did not observe any significant difference in flexural stiffnesses between intra-dimer interfaces of GTP and GDP-tubulins. Instead, the nucleotide dramatically affected the inter-dimer interface around the exchangeable nucleotide binding site. This inter-dimer interface was not examined in the former study, which was focused on free tubulin dimers. We do not think that the disagreement in our conclusions about tubulin dimers could be related to the tubulin structures, or molecular dynamics simulation parameters we used, as they were similar. But there were differences in the approach, with which the flexibilities were assessed. Here we measured the flexibility of tubulin interfaces around their relaxed curved conformations, while Igaev and Grubmüller used the umbrella sampling method to probe energetic landscape on a larger scale along a reaction coordinate, which did not exactly correspond to the bend and twist angles, which we used in this study. An equipartition-based analysis, similar to ours, was first carried out in the pioneering paper by Grafmüller and Voth [15]. In contrast to the present study, the authors did not find any statistically significant nucleotide-dependence of either intra- or inter-tubulin interfaces. The stiffness of inter-dimer interfaces clearly depended on the structures they used. At the time of the study, only a low resolution structure of straight GDP-bound tubulin from Zn-induced sheets with antiparallel protofilaments was available (PDB code: 1JFF). That tubulin structure was compacted around the exchangeable nucleotide-binding site, so it is unclear if simple insertion of Mg^2+^ ion and substituting GDP molecule for GTP would drive a correct GTP-tubulin conformation, given the complexity of tubulin’s energy landscape. The authors also examined the relaxation of tubulins from the curved structure (PDB code: 1SA0), which represented a complex of colchicine, stathmin and GDP-tubulin tetramer. Although the structure had a higher resolution, the interface between dimers in presence of stathmin could be affected by this MAP. Moreover, computational resources now allow substantially longer simulation times, which increase the chances of more complete relaxation of the tubulin structure.

**Fig 7:**
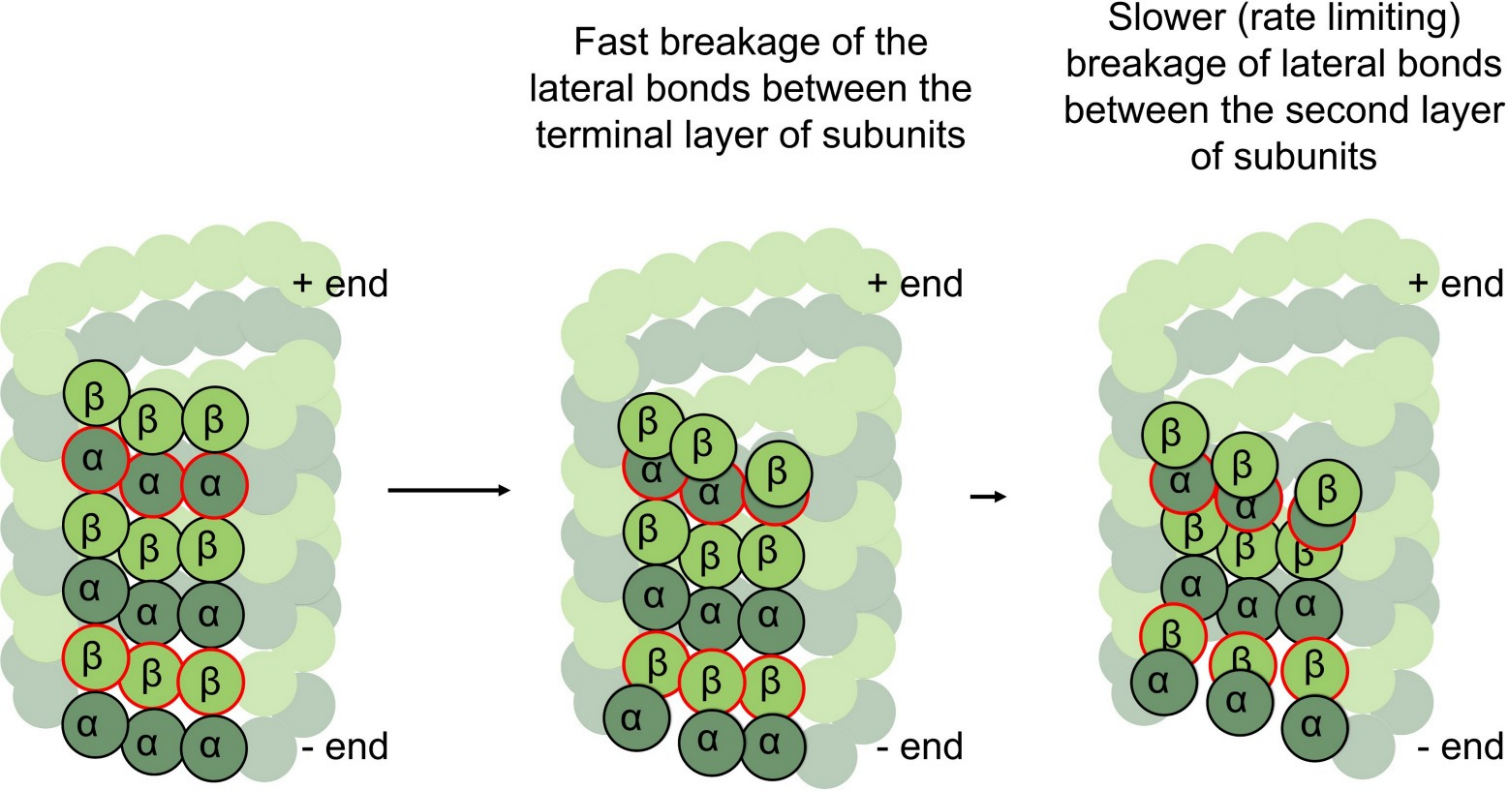
Implications of unequal interface stiffness and non-radial bending for dynamic properties of plus- and minus- microtubule tips. Three tubulin protofilaments are highlighted. α-tubulins are dark green, and β- tubulins are light green. Red outlines mark the second to terminal layers of tubulins. In the GTP-state of tubulins those layers are under lower bending stress, and so they rate limit assembly-disassembly at plus- and minus-ends of the microtubule.

Finally, we find unequal flexural stiffness of the inter-dimer and intra-dimer interfaces of GTP-tubulins, and less different but still distinct flexural stiffness of inter- and intra-dimer interfaces of GDP-tubulins. We argue that these features could be essential for explaining the difference in the rates of assembly and disassembly of plus- and minus-ends of microtubules. It has recently been proposed, that microtubules assemble and disassemble by dynamic peeling and unpeeling of curved GTP-protofilaments at the microtubule tip, so that the balance between the lateral bonds and outward bending/twisting energy controls the rate of both microtubule assembly and disassembly [14]. The minus-ends of microtubules terminate with α-tubulins, while the plus-ends terminate with β-tubulins. Obviously, if the lateral bonds between α-α and β-β tubulins were equal, microtubule rates would be identical at both ends. Distinct lateral bonds between α-α and β-β tubulins alone cannot render the rates different either, if the flexural stiffnesses between any pair of tubulin subunits is the same. However, if the inter-dimer interface is soft, compared to the intra-dimer interface, the terminal layer of subunits is under higher bending stress compared to the layer second to terminal (Fig 7). Indeed, the lateral bonds of the terminal layer are opposed by the large bending force, coming from stiff intra-dimer interface, trying to curve the subunit out and break the lateral bonds, so these layers should break lateral bonds relatively fast. The layer second to terminal, on the other hand, is under lower bending stress, because the inter-dimer stiffness is significantly softer. Therefore, lateral bonds holding the second to terminal layers would exist for longer time and become rate limiting. This means that the second to terminal layer, composed of β-tubulins at the minus-end and α-tubulins at the plus-end can control the rates of assembly from GTP-tubulin, explaining why those rates could differ.

An alternative but not mutually exclusive explanation of the difference in plus- and minus-end assembly/disassembly rates, obviously, is a possible difference in the on-rates of tubulins associating with microtubules at either end. However, it is unclear how such difference in the on-rates could arise, as association at either microtubule end seems to be dependent on the same kinds of interactions.

Overall, our study highlights a set of key mechanical properties of tubulin interfaces, shedding new light onto the mechanism of microtubule dynamics. We hope that our results will facilitate construction of more complete and realistic models of microtubule dynamics in the future.

## Methods

### Molecular dynamics simulations

Molecular models of the straight GDP-bound tubulin and Mg-GTP-bound tubulin structures (dimers, tetramers and hexamers) were based on 3J6F and 3J6E PDB structures [22]. The latter contained the slowly hydrolysable GTP analog (GMPCPP) in the exchangeable nucleotide-binding site of β-tubulin. GMPCPP was converted into GTP by replacing the carbon atom between α- and β-phosphate with an oxygen atom, and the new bond lengths and angle relaxed to their equilibrium values during minimization. Molecular model of three laterally bonded tubulins was based on 3J6F PDB structure. We added unresolved mobile amino acid chains in all the models, using the Modeller program [47]. Propka software [48] was used to calculate the unknown degree of protonation of ionizable amino acid residues and Dowser program [49] was used to identify and solvate cavities inside the protein. A virtual three-dimensional cubic reaction volume filled with TIP3P water with periodic boundary conditions was used for the simulation. The size of the reaction volume was set in such a way that the distance from the protein surface to the nearest box boundary was not initially less than two nanometers. The ionic strength of the solution was set at 100 mM by adding K^+^ and Cl^−^ ions and the total charge of the system was zero. Simulations were performed using the GROMACS 5 software package, which allows parallel computing on hybrid architecture [50] with the CHARMM27 force field [51,52]. The parameters of the GTP and GDP molecules were also taken from the CHARMM27 force field, and parameters of their phosphate groups were set in accordance with [53], similar to the phosphate groups of ATP and ADP.

After preparing each of the tubulin systems as described above, we minimized their energies using the steepest descent algorithm. Energy minimization was followed by a two-step equilibration. First, we conducted one-nanosecond-long simulations with constrained positions of all heavy protein atoms at constant pressure and temperature. Second, we carried out five-nanoseconds-long simulations with constrained positions of protein backbone atoms, using the Berendsen barostat (time constant 4.0 fs, compressibility 4.5 × 10^−5^ bar^−1^) and the Berendsen thermostat. The production simulation runs were carried out in the NPT ensemble at 300K, using the Parrinello-Rahman algorithm [54] and the V-rescale thermostat for a duration of 1 μs each. In the simulations with single and triple tubulin hexamer systems position restrain was applied on all Cα atoms of the minus-end proximal α-tubulin subunits. The particle mesh Ewald method was used to treat the long-range electrostatics. All-bond P-LINCS constraints and mass rescaling (partial transfer of mass from heavy atoms to bound hydrogens [55]) allowed molecular dynamics simulations with 4 fs time step. VMD [56] and Pymol (The PyMOL Molecular Graphics System, Version 2.0 Schrödinger, LLC) were used for visualization. Detailed analysis of computational performance of simulations is presented in [57].

### Analysis of tubulin conformational changes at the interface between monomers

Analyses of bend and twist angles at tubulin interfaces were carried out with Pymol (The PyMOL Molecular Graphics System, Version 2.0 Schrödinger, LLC.) software in combination with home-made python scripts, available upon request. The scripts were realizing the following procedure. First, a coordinate system was associated with a fragment of microtubule wall structure (PDB codes: 3J6E and 3J6F), so that the orientations of the coordinate vectors were such as illustrated in Fig 1A. Then we aligned the pair of tubulin monomers under examination onto the microtubule wall fragment. For the alignment we used only the terminal globular domains of one of the tubulin subunits in the examined pair. The terminal domains were defined as described previously [15]. This way the reference subunit was aligned along the microtubule-bound coordinate system *xyz* (Fig 1B, blue). To determine the orientation of the second tubulin subunit in the examined pair relative to the reference subunit, another microtubule wall fragment was aligned onto the second tubulin subunit, producing three more orientation vectors: *X*, *Y*, *Z* (Fig 1B, red). The magnitude *θ* and direction *φ* of the bending at the tubulin-tubulin interface were calculated as:

θ=arccos(zZ) (2)

$$\varphi =\left\{ \begin{array}{c} \arccos\left(\frac{-z_{y}}{\sqrt{1-z_{z}^{2}}}\right),\;if\;Z_{x}>0\\ -arccos(\frac{-z_{y}}{\sqrt{1-z_{z}^{2}}}),\;if\;Z_{x}\leq 0 \end{array}\right.$$ (3)

Further, auxiliary coordinate vectors, ***x'***, ***y'***, ***z'***, shown in Fig 1B in cyan, were obtained by rotating the coordinate vectors ***x*, *y*, *z*** by angle *θ* around axis ***P***. That axis was defined as cross-product of vectors ***z*** and ***Z***. Amplitude of the twist angle was calculated as:

δ=arccos(x′X) (4)

The sign of the *δ*-angle was defined positive when the triple product of vectors {***x'***, ***X***, ***Z***} was positive. In other words, the clockwise direction (viewed from the microtubule plus-end), was defined as positive twist, the anti-clockwise rotation was considered to be a negative twist. Thus, the conformational angles could adopt values in the following ranges: *θ* adopted values from 0 to 90 degrees; *φ* and *δ* adopted values from -180 degrees to 180 degrees, unless stated otherwise.

### Assessment of compaction of tubulin interfaces and counting contacts at the lateral and longitudinal interfaces

Compaction at tubulin interfaces was characterized with inter-tubulin domain distances[23], which were measured between centers of mass of ribose rings in the nucleotides of adjacent tubulin subunits for every nanosecond frame of the last 500 ns of each simulation and then averaged over all the runs. Only the minus-end proximal intra-dimer interfaces of tetramers were considered, as the nucleotides at the plus-ends of tetramers were more mobile and in one case even escaped tubulin pocket by the end of one-microsecond simulation.

The number of contacts between tubulins at the longitudinal interfaces was determined for every nanosecond frame of the last 500 ns of each simulation run and then averaged over all the runs. A contact was defined as proximity of Cα atoms of two amino acids closer than the 8Å threshold. Lateral contacts between pairs of terminal β-tubulin subunits of the laterally bonded hexamers were determined analogously for every nanosecond of the simulation and plotted against simulation time.

### Principal component analysis

We analyzed global modes of macromolecular mobility of tubulin oligomers using principal component analysis (PCA) applied to obtained molecular dynamics trajectories. PCA is a statistical technique commonly used for dimensionality reduction and determination of a subset of linearly independent variables (called *principal components*, *PCs*) explaining most of the variation observed in the original data. PCA relies on construction and diagonalization of the symmetric covariance matrix *C* between all the pairs of coordinates (e.g., Cartesian coordinates of atoms):

$$C=\left[\begin{array}{ccc} 1 & \cdots & \langle \left(x_{i}-\langle x_{i}\rangle )(x_{j}-\langle x_{j}\rangle \right)\rangle \\ \vdots & \ddots & \vdots \\ \langle \left(x_{j}-\langle x_{j}\rangle )(x_{i}-\langle x_{i}\rangle \right)\rangle & \cdots & 1 \end{array}\right]$$ (5)

where, *x*_*i*_ is a Cartesian coordinate of *i*-th atom, and < …> means averaging over all of the sampled conformations (e.g., frames of molecular dynamics trajectory). Diagonalization of *C* yields eigenvectors with corresponding eigenvalues. While the former represent the collective motions, the latter designate the respective variance. In case of molecular simulations, a limited number of eigenvectors (usually 1–10) with the largest eigenvalues describe the vast majority of variance [58].

PCA was performed with the Prody toolkit [59] using protein C_α_ atoms only (excluding flexible loops and C-terminal tails) and a coarse-grained representation for GTP/GDP (one bead per guanine, ribose, each phosphate group and magnesium) upon the roto-translational alignment of all the frames of molecular dynamics trajectories to the initial configurations.

### Normal mode analysis, parametrization of the elastic networks and estimation of stiffness

Normal mode analysis (NMA) is a powerful and widely used method for prediction of functional modes of protein mobility [60] and assessment of mechanical properties of macromolecular complexes [37]. This approach is computationally similar to PCA, but instead of covariance matrix, the Hessian matrix (composed of the second-order derivatives of the potential energy by coordinates) is diagonalized, giving eigenvectors that correspond to the collective, low-frequency molecular modes, and eigenvalues that characterize the stiffness of the corresponding normal modes. Due to the large size of the systems investigated, we used NMA in its simplified form, based on the elastic network model (ENM). The built ENM was based on the straight conformations of tubulin tetramers and consisted of protein C_α_ atoms and a coarse-grained representation for GTP/GDP (akin to a subset of atoms used in PCA) connected with harmonic springs when closer than the cutoff distance, *R*_*cutoff*_ = 0.8 nm. The beads were assigned the masses of all-atom fragments replaced by them. Instead of uniform spring constants normally used for ENM, we parametrize heterogeneous ENM based on the performed all-atom simulations, which results in more accurate models [61]. We used the parametrization procedure based on the Boltzmann inversion and the iterative scheme, which we describe in details in [62]. In brief, the approach is based on fitting the fluctuations of pair distances in ENM to corresponding fluctuations computed from all-atom simulations via iterative adjustment of spring constants in ENM.

The heterogeneous ENM analysis was performed by in-house scripts exploiting the Prody toolkit [59]. The collective modes acquired by NMA were compared with principal components obtained for the same systems by computing their pairwise overlaps, which are given by the correlation cosines of corresponding eigenvectors [63].

The eigenvalue of *n*-th normal mode, *λ*_*n*_, is related to its angular frequency, $\omega_{n}=\sqrt{\lambda_{n}}$. The related vibrational frequency and mechanical stiffness for each normal mode of tubulin oligomers can be estimated applying the linear elastic beam theory [37,38]. In our analysis we assumed that tubulin oligomers behave as freely vibrating elastic filaments. Then, bending stiffness *K*_*bend*_ (for normal modes corresponding to bending of oligomers) and torsional rigidity *K*_*twist*_ (for normal modes resembling twist-like motions of oligomers) can be found from equations:

$$K_{bend}=\frac{\rho_{l}\omega^{2}}{k^{4}}$$ (6)

and

$$K_{twist}=\frac{\rho_{v}I\omega^{2}}{k^{2}}$$ (7)

where *ω*—angular frequency, *k*—corresponding wavenumber (depending on the specific boundary conditions of the wave equations and taken from [36]), *ρ*_*l*_—mass per unit length (1.95⋅10^−14^ kg/m), *ρ*_*v*_—mass per unit volume (1.3⋅10^3^ kg/m^3^), and *I* –the moment of inertia of the cross-sectional area with respect to the long axis of the filament (3.42⋅10^−35^ m^4^).

## Supporting information

S1 Fig Bending and twisting of tubulin interfaces in other published tubulin structures. (A) Time-dependence of *φ*, *θ*, and *δ*-angles, describing a simulation of GDP-tubulin dimer, based on 1JFF PDB structure. (B) Projections of the unit OZ-vector of the β-subunit of 1JFF-tubulin dimer interface onto xy-plane of the α-tubulin at every ns of the simulation after the first 500 ns. Data and color-coding correspond to panel A. Dashed line schematically shows the circumference of the microtubule. Horizontal axis is tangential to the microtubule, Vertical axis is directed radially toward microtubule axis. (C) Projections of the unit OZ-vector of the upper subunit of published structures onto xy-plane of the lower-tubulin at each interface. Colors mark different structures (also see Table 2). Intra-dimer interfaces are shown with circles. Inter-dimer interfaces are shown as diamonds. The crystal structures were selected to represent diverse examples of tubulin complexes with MAPs.
(TIF)

S2 Fig Fraction of the total variance explained by the principal components. PCA was performed for the joint GTP- and GDP-trajectories of whole tetramers (A), inter-dimer interface (B) and intra-dimer interface (C).
(TIFF)

S3 Fig PCA analysis of intra-dimer interface in GTP- and GDP-tetramers. Projection of GTP- and GDP-tetramer trajectories (only last 500 ns of each simulation were used for the analysis) onto the first two PCs, obtained for the joined ensemble consisting of the GTP- and GDP-trajectories of intra-dimer interface. Probability densities for PC1 and PC2 are shown along the corresponding axes and they are constructed using Gaussian kernel density estimation.
(TIF)

S4 Fig PCA analysis of GTP- and GDP-tetramers. PCA analysis of inter-dimer interface in GTP- and GDP-tetramers. Projection of GTP- and GDP-tetramer trajectories (only last 500 ns of each simulation were used for the analysis) onto the first two principal components obtained for the joined ensemble consisting of the GTP- and GDP-trajectories of inter-dimer interface. Probability densities for PC1 and PC2 are shown along the corresponding axes and they are constructed using Gaussian kernel density estimation.
(TIF)

S5 Fig Time dependence of conformational changes in the simulations of tubulin dimers. (A) Time-dependence of *φ*, *θ*, and *δ*-angles, describing GDP-tubulin dimer bending direction, bending magnitude and magnitude of twist, respectively. Colors mark two independent simulation runs. (B) Time-dependence of *φ*, *θ*, and *δ*-angles for GTP-tubulin dimer interface in two independent simulations (shown in red and green).
(TIF)

S6 Fig Time-dependence of direction of tubulin bending (*φ*) in simulations of tubulin tetramers. Time-dependence of *φ-*angles, describing: (A) intra-dimer GDP-tubulin bending direction of tubulin tetramers; (B) intra-dimer GTP-tubulin bending direction of tubulin tetramers; (C) inter-dimer GDP-tubulin bending direction of tubulin tetramers; (D) inter-dimer GTP-tubulin bending direction of tubulin tetramers. Colors mark independent simulation runs and correspond main Figs 3 and 4. Note that high magnitude of *φ-*angle fluctuations is often related to the low magnitude of bending (*θ-*angle in Figs 3 and 4), which means that the direction of bending is poorly defined.
(TIF)

S7 Fig Convergence of the bending angle for the intra- and inter-dimer interfaces of tubulin tetramers. The last 500 ns of each trajectory (shown in different colors: red, yellow and blue) were split in four datasets (each 125 ns long) and separate box plots for the bending angle were evaluated.
(TIF)

S8 Fig Comparison of protofilament bending in the simulations with a single and three tubulin hexamers. Smoothed projections of the centers of mass of plus-end-proximal β-tubulin subunits onto XY plane of microtubule-bound coordinate system are shown in each case.
(TIF)

S1 Table Overlap between PC of the molecular dynamics simulations of tubulin dimers and respective NMs. The fraction of explained total variation is given in parentheses for each PC.
(DOCX)

S2 Table Overlap between PC of the molecular dynamics simulations of tubulin tetramers and respective NMs. The fraction of explained total variation is given in parentheses for each PC.
(DOCX)

S3 Table Tubulin compaction, characterized with intra- and inter-dimer distances. (DOCX)

S4 Table PCA of the intra-dimer interface of GTP- and GDP-tetramers. Mean values and standard deviation for projections of GTP- and GDP-trajectories (only last 500 ns of each simulation were used for the analysis) onto the first two PCs.
(DOCX)

S5 Table PCA of the inter-dimer interface of GTP- and GDP-tetramers. Mean values and standard deviation for projections of GTP- and GDP-trajectories (only last 500 ns of each simulation were used for the analysis) onto the first two PCs.
(DOCX)

S6 Table Number of inter- and intra-dimer contacts calculated for the whole GTP tetramers (3J6E) or GDP tetramers (3J6F). Standard deviation is shown along with the mean value.
(DOCX)

S1 Movie Molecular dynamics simulation of a GDP-tubulin tetramer (PDB code: 3J6F). On the left: side view. On the right: top view from the plus-end. Subunits of adjacent protofilaments are shown to place the simulated tetramer in the microtubule context. Gray arc marks the microtubule circumference. Simulated α-tubulin subunits are colored darker green, simulated β-tubulin subunits are lighter green. Initial straight structure is shown in pink. GDP molecules are shown in purple, GTP molecules are shown in orange.
(MP4)

S2 Movie Molecular dynamics simulation of a GTP-tubulin tetramer (PDB code: 3J6E). On the left: side view. On the right: top view from the plus-end. Adjacent protofilaments are shown to place the simulated tetramer in the microtubule context. Gray arc marks the microtubule circumference. Simulated alpha-tubulin subunits are colored darker green, simulated beta-tubulin subunits are lighter green. Initial straight structure is shown in pink. GTP molecules are shown in orange.
(MP4)

S3 Movie Normal modes of whole tubulin tetramers. Three lowest frequency modes are shown for GTP- and GDP-bound tubulins.
(MP4)

S4 Movie PCA modes of tubulin intra- and inter-dimer interfaces. Two main PCs are shown for each interface in side and top views. Motions are exaggerated.
(MP4)

S5 Movie Molecular dynamics simulation of a GDP-tubulin hexamer, with fixed minus-end terminal α-tubulin subunit (PDB code: 3J6F). On the left: side view. On the right: top view from the plus-end. Subunits of adjacent protofilaments are shown to place the simulated hexamer in the microtubule context. Gray arc marks the microtubule circumference. Simulated alpha-tubulin subunits are colored darker green, simulated beta-tubulin subunits are lighter green. Initial straight structure is shown in pink. GDP molecules are shown in purple, GTP molecules are shown in orange.
(MP4)

S6 Movie Molecular dynamics simulation of three GDP-tubulin hexamers, with fixed minus-end terminal α-tubulin subunits (PDB code: 3J6F). On the left: front/diagonal view. On the right: top view from the plus-end. Gray arc marks the microtubule circumference. Simulated α-tubulin subunits are colored darker green, simulated β-tubulin subunits are lighter green. GDP molecules are shown in purple, GTP molecules are shown in orange.
(MP4)
