# Supplementary figures and images for: Mechanical properties of tubulin intra- and inter-dimer interfaces and their implications for microtubule dynamic instability
Source: PLoS Comput Biol. 2019 Aug 30;15(8):e1007327. doi: 10.1371/journal.pcbi.1007327 (PMC6742422; doi:10.1371/journal.pcbi.1007327)

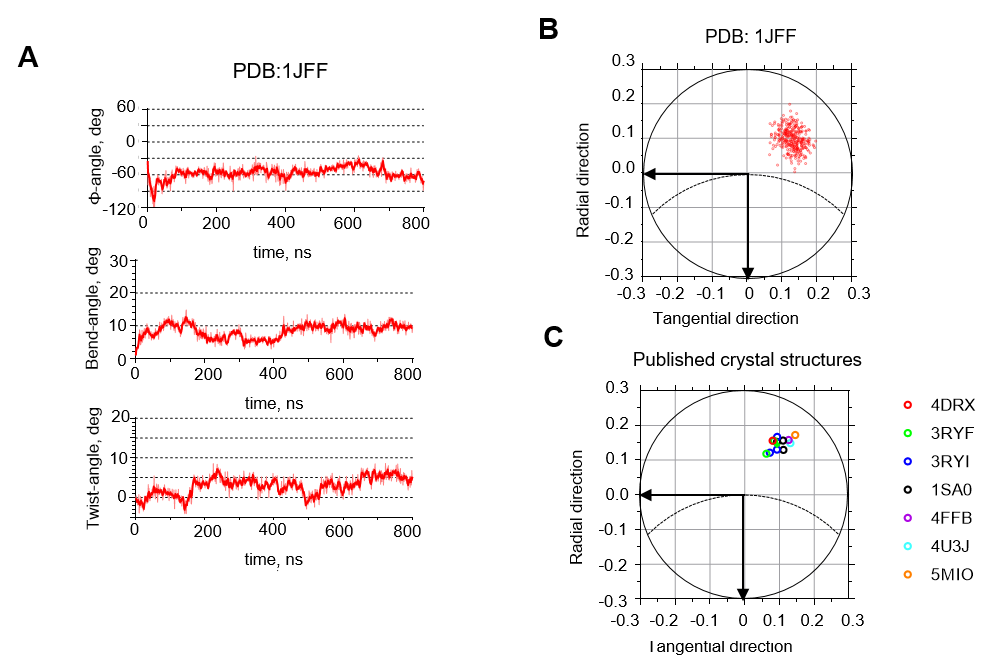

Supplement: S1 Fig — (A) Time-dependence of φ, θ, and δ-angles, describing a simulation of GDP-tubulin dimer, based on 1JFF PDB structure. (B) Projections of the unit OZ-vector of the β-subunit of 1JFF-tubulin dimer interface onto xy-plane of the α-tubulin at every ns of the simulation after the first 500 ns. Data and color-coding correspond to panel A. Dashed line schematically shows the circumference of the microtubule. Horizontal axis is tangential to the microtubule, Vertical axis is directed radially toward microtubule axis. (C) Projections of the unit OZ-vector of the upper subunit of published structures onto xy-plane of the lower-tubulin at each interface. Colors mark different structures (also see Table 2). Intra-dimer interfaces are shown with circles. Inter-dimer interfaces are shown as diamonds. The crystal structures were selected to represent diverse examples of tubulin complexes with MAPs. (TIF) [file pcbi.1007327.s001.tif]

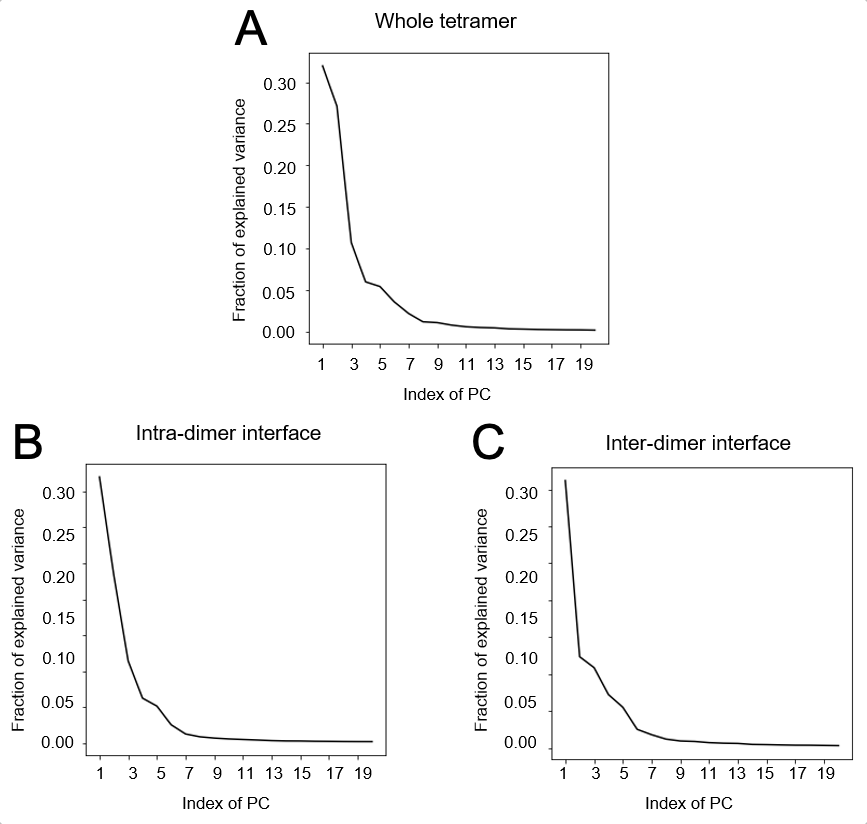

Supplement: S2 Fig — PCA was performed for the joint GTP- and GDP-trajectories of whole tetramers (A), inter-dimer interface (B) and intra-dimer interface (C). (TIFF) [file pcbi.1007327.s002.tiff]

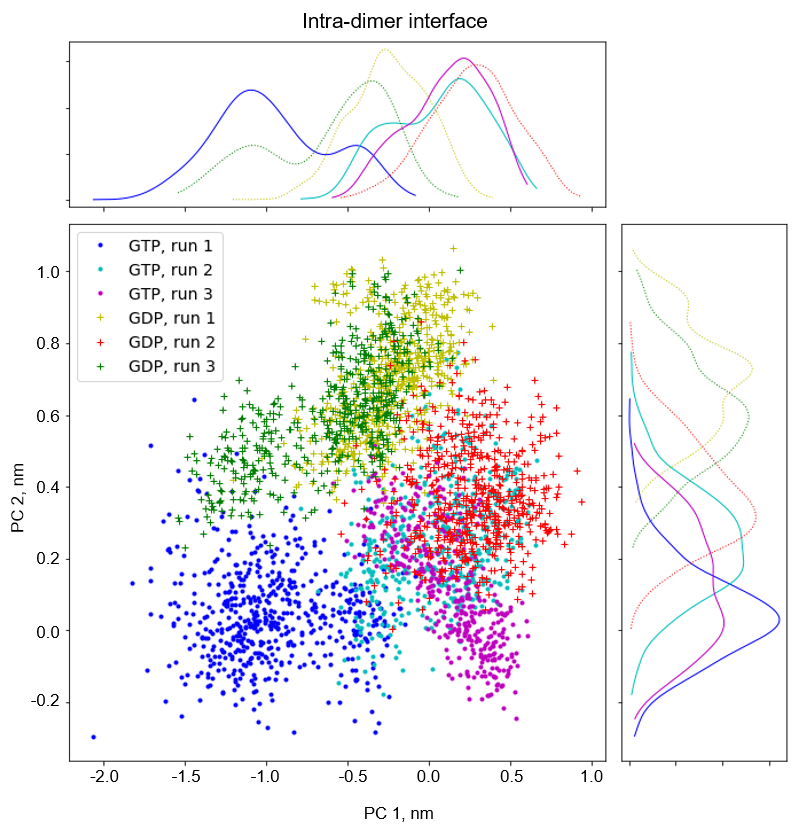

Supplement: S3 Fig — Projection of GTP- and GDP-tetramer trajectories (only last 500 ns of each simulation were used for the analysis) onto the first two PCs, obtained for the joined ensemble consisting of the GTP- and GDP-trajectories of intra-dimer interface. Probability densities for PC1 and PC2 are shown along the corresponding axes and they are constructed using Gaussian kernel density estimation. (TIF) [file pcbi.1007327.s003.tif]

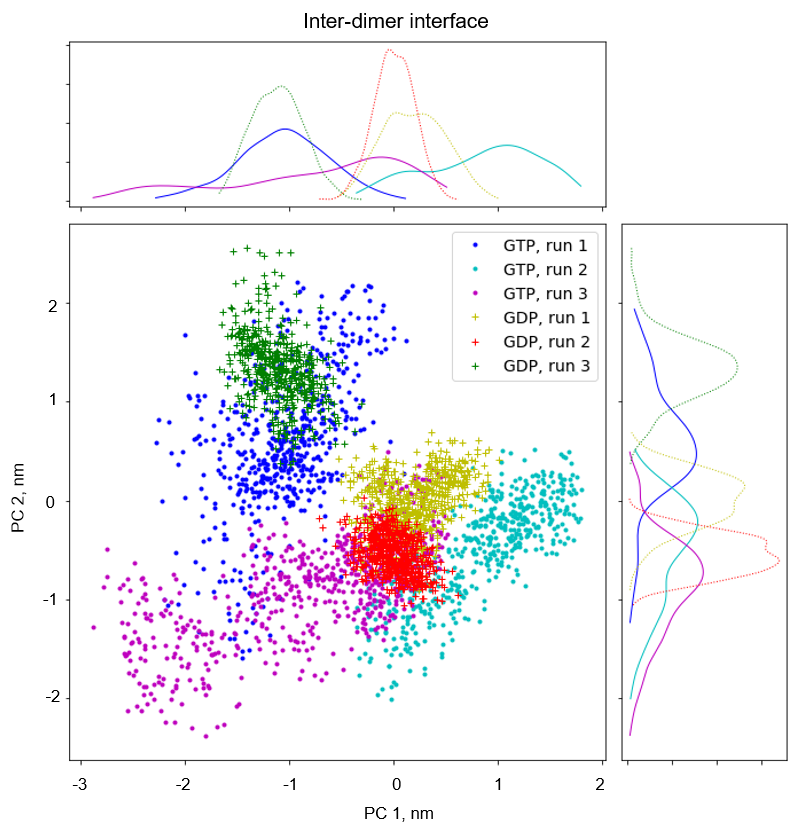

Supplement: S4 Fig — PCA analysis of inter-dimer interface in GTP- and GDP-tetramers. Projection of GTP- and GDP-tetramer trajectories (only last 500 ns of each simulation were used for the analysis) onto the first two principal components obtained for the joined ensemble consisting of the GTP- and GDP-trajectories of inter-dimer interface. Probability densities for PC1 and PC2 are shown along the corresponding axes and they are constructed using Gaussian kernel density estimation. (TIF) [file pcbi.1007327.s004.tif]

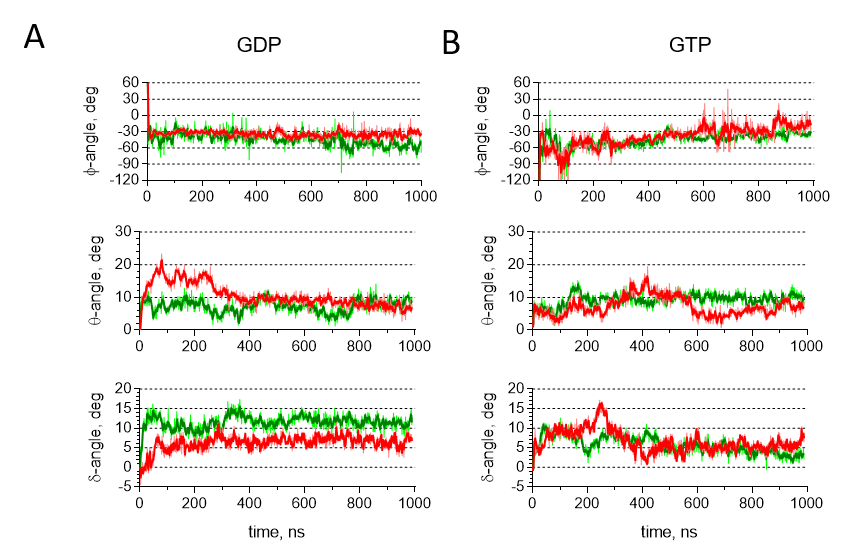

Supplement: S5 Fig — (A) Time-dependence of φ, θ, and δ-angles, describing GDP-tubulin dimer bending direction, bending magnitude and magnitude of twist, respectively. Colors mark two independent simulation runs. (B) Time-dependence of φ, θ, and δ-angles for GTP-tubulin dimer interface in two independent simulations (shown in red and green). (TIF) [file pcbi.1007327.s005.tif]

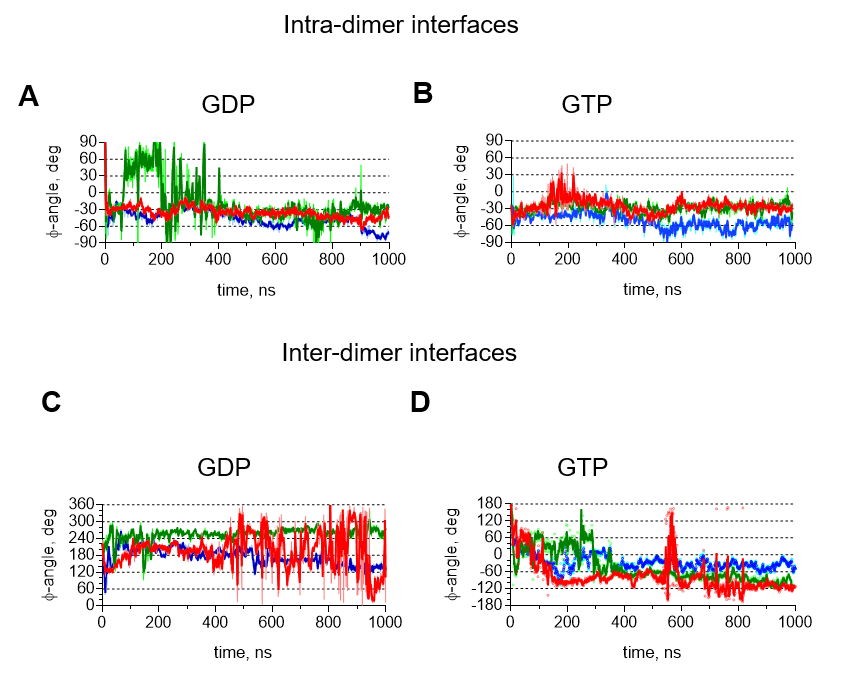

Supplement: S6 Fig — Time-dependence of φ-angles, describing: (A) intra-dimer GDP-tubulin bending direction of tubulin tetramers; (B) intra-dimer GTP-tubulin bending direction of tubulin tetramers; (C) inter-dimer GDP-tubulin bending direction of tubulin tetramers; (D) inter-dimer GTP-tubulin bending direction of tubulin tetramers. Colors mark independent simulation runs and correspond main Figs 3 and 4. Note that high magnitude of φ-angle fluctuations is often related to the low magnitude of bending (θ-angle in Figs 3 and 4), which means that the direction of bending is poorly defined. (TIF) [file pcbi.1007327.s006.tif]

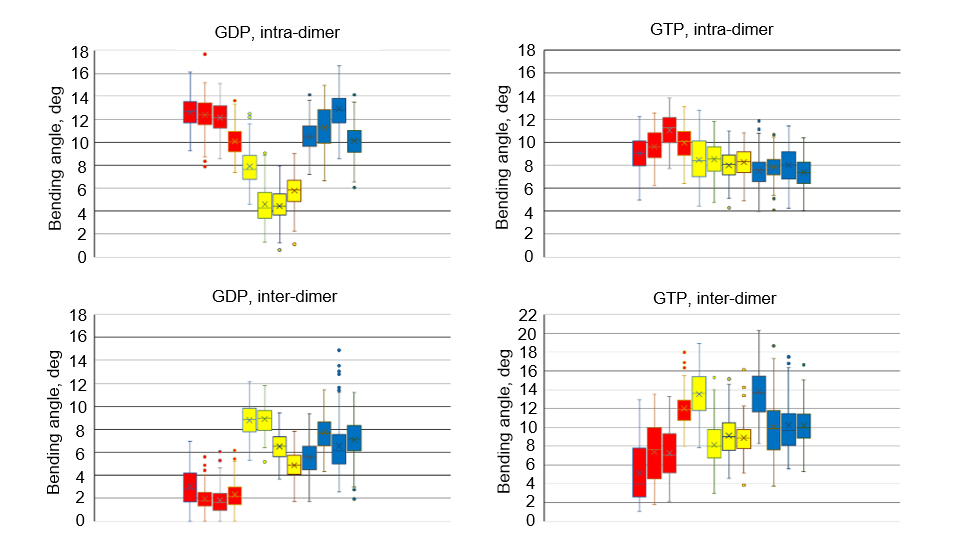

Supplement: S7 Fig — The last 500 ns of each trajectory (shown in different colors: red, yellow and blue) were split in four datasets (each 125 ns long) and separate box plots for the bending angle were evaluated. (TIF) [file pcbi.1007327.s007.tif]

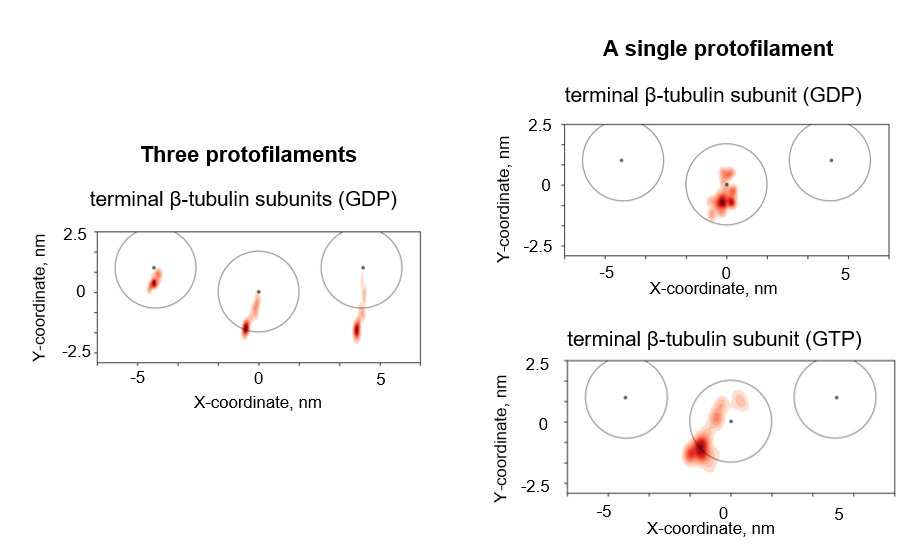

Supplement: S8 Fig — Smoothed projections of the centers of mass of plus-end-proximal β-tubulin subunits onto XY plane of microtubule-bound coordinate system are shown in each case. (TIF) [file pcbi.1007327.s008.tif]
