# Supplementary material for: Mechanical properties of tubulin intra- and inter-dimer interfaces and their implications for microtubule dynamic instability
Source: PLoS Comput Biol. 2019 Aug 30;15(8):e1007327. doi: 10.1371/journal.pcbi.1007327 (PMC6742422; doi:10.1371/journal.pcbi.1007327)
Supplement: S1 Table — The fraction of explained total variation is given in parentheses for each PC. (DOCX) [file pcbi.1007327.s009.docx]

**S1 Table.** **Overlap between PC of the molecular dynamic simulations of tubulin dimers and respective NMs.**

| Structure type and run | Principal Component (PC) | NM 1 (twist) | NM 2 (bend 1) | NM 3 (bend 2) |
| --- | --- | --- | --- | --- |
| 3j6e (run #1) | PC 1 (0.39) | 0.09 | 0.4 | 0.15 |
|  | PC 2 (0.18) | 0.6 | 0.06 | 0.27 |
|  | PC 3 (0.08) | 0.24 | 0.12 | 0.24 |
| 3j6e (run #2) | PC 1 (0.36) | 0.1 | 0.54 | 0.04 |
|  | PC 2 (0.16) | 0.53 | 0.24 | 0.05 |
|  | PC 3 (0.06) | 0.18 | 0.58 | 0.37 |
| 3j6f (run #1) | PC 1 (0.41) | 0.29 | 0.62 | 0.09 |
|  | PC 2 (0.16) | 0.39 | 0.16 | 0.4 |
|  | PC 3 (0.10) | 0.31 | 0.38 | 0.27 |
| 3j6f (run #2) | PC 1 (0.36) | 0.34 | 0.69 | 0.16 |
|  | PC 2 (0.20) | 0.48 | 0.11 | 0.11 |
|  | PC 3 (0.11) | 0.07 | 0.16 | 0.07 |
| 1jff (run #1) | PC 1 (0.39) | 0.17 | 0.41 | 0.07 |
|  | PC 2 (0.21) | 0.3 | 0.11 | 0.02 |
|  | PC 3 (0.09) | 0.35 | 0.16 | 0.11 |
