# Supplementary material for: Mechanical properties of tubulin intra- and inter-dimer interfaces and their implications for microtubule dynamic instability
Source: PLoS Comput Biol. 2019 Aug 30;15(8):e1007327. doi: 10.1371/journal.pcbi.1007327 (PMC6742422; doi:10.1371/journal.pcbi.1007327)
Supplement: S2 Table — The fraction of explained total variation is given in parentheses for each PC. (DOCX) [file pcbi.1007327.s010.docx]

**S2 Table.** **Overlap between PC of the molecular dynamic simulations of tubulin tetramers and respective NMs.**

| Structure type and run | Principal Component (PC) | NM 1 (bend 1) | NM 2 (bend 2) | NM 3 (twist) |
| --- | --- | --- | --- | --- |
| GDP tetramer 3j6f (run #1) | PC 1 (0.53) | 0.68 | 0.37 | 0.4 |
|  | PC 2 (0.20) | 0.55 | 0.73 | 0.27 |
|  | PC 3 (0.11) | 0.23 | 0.47 | 0.7 |
| GDP tetramer 3j6f (run #2) | PC 1 (0.49) | 0.83 | 0.32 | 0.31 |
|  | PC 2 (0.11) | 0.39 | 0.63 | 0.19 |
|  | PC 3 (0.08) | 0.3 | 0.02 | 0.66 |
| GDP tetramer 3j6f (run #3) | PC 1 (0.48) | 0.41 | 0.05 | 0.75 |
|  | PC 2 (0.20) | 0.07 | 0.89 | 0.04 |
|  | PC 3 (0.11) | 0.83 | 0.12 | 0.37 |
| GTP tetramer 3j6e (run #1) | PC 1 (0.54) | 0.75 | 0.56 | 0.13 |
|  | PC 2 (0.15) | 0.4 | 0.64 | 0.22 |
|  | PC 3 (0.07) | 0.39 | 0.24 | 0.67 |
| GTP tetramer 3j6e (run #2) | PC 1 (0.63) | 0.42 | 0.26 | 0.05 |
|  | PC 2 (0.13) | 0.23 | 0.59 | 0.02 |
|  | PC 3 (0.06) | 0.06 | 0.16 | 0.29 |
| GTP tetramer 3j6e (run #3) | PC 1 (0.52) | 0.66 | 0.69 | 0.02 |
|  | PC 2 (0.23) | 0.67 | 0.65 | 0.02 |
|  | PC 3 (0.10) | 0.02 | 0.04 | 0.89 |
