# Supplementary material for: Mechanical properties of tubulin intra- and inter-dimer interfaces and their implications for microtubule dynamic instability
Source: PLoS Comput Biol. 2019 Aug 30;15(8):e1007327. doi: 10.1371/journal.pcbi.1007327 (PMC6742422; doi:10.1371/journal.pcbi.1007327)
Supplement: S3 Table — (DOCX) [file pcbi.1007327.s011.docx]

**S3 Table. Tubulin compaction, characterized with intra- and inter-dimer distances**

| PDB id | Nucleotide | Intra-dimer distance, Å | Inter-dimer distance, Å | Comment |
| --- | --- | --- | --- | --- |
| 3j6f | GDP | 41.5 | 40 | initial straight structure, in the lattice |
| 3j6e | GTP | 41.3 | 41.9 | initial straight structure, in the lattice |
| 3j6f | GDP | 42.9 ± 0.6 | 41.1 ± 0.5 | average during last 500ns of simulation |
| 3j6e | GTP | 43.6 ± 0.9 | 41.5 ± 0.1 | average during last 500ns of simulation |
| 1sa0 | GDP | 43.2 ± 0.2 | 42.3 | curved structure (Stathmin, colchicine) |
