# Supplementary material for: Mechanical properties of tubulin intra- and inter-dimer interfaces and their implications for microtubule dynamic instability
Source: PLoS Comput Biol. 2019 Aug 30;15(8):e1007327. doi: 10.1371/journal.pcbi.1007327 (PMC6742422; doi:10.1371/journal.pcbi.1007327)
Supplement: S4 Table — Mean values and standard deviation for projections of GTP- and GDP-trajectories (only last 500 ns of each simulation were used for the analysis) onto the first two PCs. (DOCX) [file pcbi.1007327.s012.docx]

**Table S4.** **PCA of the intra-dimer interface of GTP- and GDP-tetramers.**

| Structure type and run | Mean (PC1), nm | SD (PC1), nm | Mean (PC2), nm | SD (PC2), nm |
| --- | --- | --- | --- | --- |
| GTP, 3j6e (run #1) | -0.96 | 0.36 | 0.05 | 0.14 |
| GTP, 3j6e (run #2) | 0.06 | 0.29 | 0.25 | 0.15 |
| GTP, 3j6e (run #3) | 0.12 | 0.25 | 0.13 | 0.17 |
| GDP, 3j6f (run #1) | -0.24 | 0.24 | 0.7 | 0.15 |
| GDP, 3j6f (run #2) | 0.27 | 0.26 | 0.38 | 0.14 |
| GDP, 3j6f (run #3) | -0.61 | 0.38 | 0.6 | 0.15 |
