# Supplementary material for: Mechanical properties of tubulin intra- and inter-dimer interfaces and their implications for microtubule dynamic instability
Source: PLoS Comput Biol. 2019 Aug 30;15(8):e1007327. doi: 10.1371/journal.pcbi.1007327 (PMC6742422; doi:10.1371/journal.pcbi.1007327)
Supplement: S5 Table — Mean values and standard deviation for projections of GTP- and GDP-trajectories (only last 500 ns of each simulation were used for the analysis) onto the first two PCs. (DOCX) [file pcbi.1007327.s013.docx]

**Table S5.** **PCA of the inter-dimer interface of GTP- and GDP-tetramers.**

| Structure type and run | Mean (PC1), nm | SD (PC1), nm | Mean (PC2), nm | SD (PC2), nm |
| --- | --- | --- | --- | --- |
| GTP, 3j6e (run #1) | -1.05 | 0.44 | 0.59 | 0.61 |
| GTP, 3j6e (run #2) | 0.85 | 0.54 | -0.51 | 0.54 |
| GTP, 3j6e (run #3) | -0.75 | 0.87 | -0.87 | 0.52 |
| GDP, 3j6f (run #1) | 0.2 | 0.3 | 0 | 0.28 |
| GDP, 3j6f (run #2) | 0.01 | 0.2 | -0.55 | 0.2 |
| GDP, 3j6f (run #3) | -1.11 | 0.25 | 1.34 | 0.35 |
