# Supplementary material for: Mechanical properties of tubulin intra- and inter-dimer interfaces and their implications for microtubule dynamic instability
Source: PLoS Comput Biol. 2019 Aug 30;15(8):e1007327. doi: 10.1371/journal.pcbi.1007327 (PMC6742422; doi:10.1371/journal.pcbi.1007327)
Supplement: S6 Table — Standard deviation is shown along with the mean value. (DOCX) [file pcbi.1007327.s014.docx]

**S6 Table. Number of inter- and intra-dimer contacts calculated for the whole GTP tetramers (3j6e) or GDP tetramers (3j6e).**

| **Structure type and run** | **Intra-dimer contacts**  **(averaged over 2 interfaces)** | **Inter-dimer contacts** |
| --- | --- | --- |
| GDP tetramer (3j6f), run #1 | 59.8 ± 3.1 | 80.8 ± 5.6 |
| GDP tetramer (3j6f), run #2 | 54.4 ± 2.8 | 72.2 ± 5.6 |
| GDP tetramer (3j6f), run #3 | 55.5 ± 3.3 | 85.0 ± 7.2 |
| GTP tetramer (3j6e), run #1 | 52.9 ± 3.8 | 47.3 ± 6.1 |
| GTP tetramer (3j6e), run #2 | 60.1 ± 3.3 | 69.7 ± 6.2 |
| GTP tetramer (3j6e), run #3 | 56.6 ± 3.3 | 53.8 ± 6.4 |
